# Multilocus phylogeny- and fruiting feature-assisted delimitation of European *Cyclocybe aegerita* from a new Asian species complex and related species

**DOI:** 10.1007/s11557-020-01599-z

**Published:** 2020-10-08

**Authors:** Roman A. Frings, Jose G. Maciá-Vicente, Sandra Buße, Adéla Čmoková, Harald Kellner, Martin Hofrichter, Florian Hennicke

**Affiliations:** 1 Junior Research Group Genetics and Genomics of Fungi, Department of Geobotany, Ruhr-University Bochum (RUB), Bochum, Germany; 2 Institute of Ecology, Evolution & Diversity, Goethe-University, Frankfurt/M., Germany; 3 Department of Botany, Charles University Prague, Prague, Czech Republic; 4 Technische Universität Dresden, International Institute (IHI) Zittau, Zittau, Germany

**Keywords:** Basidiomycota, Mushroom biogeography, Fruiting, Molecular systematics

## Abstract

*Cyclocybe aegerita* (synonym: *Agrocybe aegerita*) is a widely cultivated edible and reportedly almost cosmopolitan mushroom species that serves as a model fungus for basidiome formation and as producer of useful natural products and enzymes. Focusing on strains from different continents, here, we present a phylogenetic analysis of this species and some adjacent taxa that employs four phylogenetic markers. In addition, we tested the strains’ capability to fructify on agar media. Our analysis reveals that “*C. aegerita sensu lato*” splits up into the following two well-supported monophyletic geographic lineages: a European clade and an Asian clade. The European one is closely associated with the Chinese species *Cyclocybe salicaceicola*. In contrast, the Asian lineage, which we preliminarily designate as *Cyclocybe chaxingu* agg., may comprise several species (species complex) and clusters with the Pacific species *Cyclocybe parasitica* (New Zealand). In addition, fruiting properties differ across *C. aegerita* and its Asian and Pacific relatives; however, strains from the Asian clade and *C. parasitica* tend to form larger basidiomes with relatively big caps and long stipes and strains from the European clade exhibit a more variable fruiting productivity with the tendency to form more basidiomes, with smaller caps and shorter stipes. Moreover, some strains showed individual fruiting patterns, such as the preference to fruit where they were exposed to injuring stimuli. In conclusion, the delimitation of the newly delimited Asian species complex from our multilocus phylogeny of “*C. aegerita sensu lato*”, which is supported by phenotypic data, depicts an exemplary case of biogeographic diversity within a previously thought homogeneous species of near worldwide distribution.

**Electronic supplementary material:**

The online version of this article (10.1007/s11557-020-01599-z) contains supplementary material, which is available to authorized users.

## Introduction

The Black Poplar Mushroom *Cyclocybe aegerita* (V. Brig.) Vizzini (synonym: *Agrocybe aegerita* (V. Brig.) Singer) is an agaric that causes a moderate white-rot by chiefly degrading dead wood of deciduous trees, especially in *Populus* and *Salix* spp. (Esser et al. 1974; Nauta 2005; Uhart and Albertó 2007). With respect to its practical usage, *C. aegerita* represents an important fungal species cultivated as a choice edible mushroom in many countries, which fruits in consecutive flushes on its spawn substrate (Uhart et al. 2008). It also serves as a model basidiomycete to study basidiome (fruiting body, basidiocarp, mushroom) formation (Esser et al. 1974; Labarère and Noël 1992; Herzog et al. 2016) and to produce biotechnologically relevant enzymes of the UPO-type (unspecific peroxygenases, EC 1.11.2.1; Hofrichter et al. 2015, 2020), as well as a source of useful natural products like bioactive terpenoids and ribotoxins (Zhao et al. 2003; Ngai et al. 2005; Kögl et al. 2007; Hennicke et al. 2019; Surup et al. 2019; Tayyrov et al. 2019). By using the genome sequence of *C. aegerita* AAE-3 (Gupta et al. 2018), a molecular genetic toolset has recently been developed, now allowing functional genetic approaches to this fungus (Herzog et al. 2019).

Based on single-locus DNA sequence information coding for ribosomal RNA gene and spacer regions, it was shown that – in contrast to the type species of the genus *Agrocybe* Fayod, *Agrocybe praecox* that is associated to the Strophariceae Singer and A.H. Sm. 1946 – *C. aegerita* is closer to members of the Tubariaceae Vizzini 2008 (He et al. 2019). Thus, it has been moved from *Agrocybe* to the resurrected genus *Cyclocybe* Velen. (Vizzini et al. 2014), its currently valid name being *Cyclocybe aegerita* (V. Brig.) Vizzini (Nauta 2005; Vizzini et al. 2014; He et al. 2019; Surup et al. 2019). Apart from *C. aegerita*, Vizzini et al. (2014) also moved four other *Agrocybe* species to *Cyclocybe* because of their relatedness to *C. aegerita* based on their rDNA data. This includes the Chinese species *Agrocybe chaxingu* Huang (Zhi 1991), a cultivated mushroom in East Asia, which Vizzini et al. (2014), however, not least due to interfertility of a Chinese strain with a French one (Callac et al. 2011), discussed to potentially just represent a morphotype of the European species. In consequence, here, we address the species complex a priori by the term “*C. aegerita sensu lato*”. Reclassification into *Cyclocybe* was also applied to the species *Cyclocybe salicaceicola* (Zhu L. Yang, M. Zang & X.X. Liu) Vizzini and the Pacific species *Cyclocybe parasitica* (G. Stev.) Vizzini (Vizzini et al. 2014). The former species was described from Yunnan (China), morphologically differing from *C. aegerita* by a pale-coloured pileus, decurrent lamellae, and a lack of chlamydospore production in artificial culture according to Yang et al. (1993). The latter species, *C. parasitica*, was originally described from New Zealand as a pathogen of the plant genera *Plagianthus* and *Hoheria* (Stevenson 1982). Eventually, Vizzini et al. (2014) also reclassified *Agrocybe erebia* (Fr.) Kühner ex Singer into *Cyclocybe* (as *Cyclocybe erebia* (Fr.) Vizzini & Matheny), a plant litter-/soil-dwelling species, which was once grouped – along its wood-decaying relative *C. aegerita* – into the subgenus *Aporus* Singer. Members of this subgenus of *Agrocybe* exhibit basidiospores with an absent or inconspicuous germ pore (Nauta 2005). Furthermore, *C. aegerita* and *C. erebia*, form basidiomes with a well-developed annulus, and, according to phylogenetic analyses based on single ribosomal RNA gene and spacer region sequence data by Vizzini et al. (2014), they ought to be closely related to each other, and to *Cyclocybe erebioides* Angelini & Vizzini.

Gupta et al. (2018) first hypothesized that *C. aegerita* strains from different continents of this reportedly almost cosmopolitan fungus (Labarère and Noël 1992; Stamets 1993; Nauta 2005; Roca et al. 2009) may differ from each other. In this context, a clarifying comprehensive phylogenetic analysis of strains of “*Cyclocybe aegerita sensu lato*” from different continents as well as of other *Cyclocybe* species, based on sequence information additional to the one of single ribosomal RNA gene and spacer regions, such as the protein-coding genes *RPB2* and *TEF1α* (Matheny et al. 2006), is still lacking. Such an approach is the more required since intragenomic heterogeneity of spacer regions has been reported to be much more variable than the average 0.1–3% (Smith et al. 2007; Simon and Weiss 2008; Kovács et al. 2011; Vydryakova et al. 2012) in prominent Agaricomycotina taxa, such as *Amanita* and *Laetiporus* where 10–15% variability was recorded (Lindner and Banik 2011; Hughes et al. 2018).

Fructification of *C. aegerita* on artificial media has been repeatedly achieved with diverse strains in different settings (Esser et al. 1974; Labarère and Noël 1992; Uhart and Albertó 2007; Uhart et al. 2008; Herzog et al. 2016). Thus, a characterization of fruiting properties of geographically distant strains of different *Agrocybe* and *Cyclocybe* species in a standardized fruiting setup, such as the one established by Herzog et al. (2016), may complement a comprehensive phylogenetic analysis on this species complex, by providing additional morpho-physiological characteristics for species delimitation.

Thus, the aim of this work was to provide such a phylogenetic analysis including close relatives of “*C. aegerita* s.l.”, i.e., *C. erebia*, *C. parasitica*, and *C. salicaceicola*, and a robust assessment on their relatedness to *C. aegerita*.

## Material and methods

### Strains, culture maintenance, and assessment of fruiting characteristics

For culture maintenance, *Agrocybe* and *Cyclocybe* spp. strains were routinely propagated on 2% (*w*/*v*) malt extract agar (MEA; 70167-500G, Sigma-Aldrich Chemie GmbH Munich, Germany). For fruiting, 1.5% MEA was used. All strains tested under the fruiting induction regime of Herzog et al. (2016) in this study are listed in Table 1. Despite of requests for strains of the Chinese species *C. salicaceicola* (Zhu L. Yang, M. Zang & X.X. Liu) Vizzini from the authors Chen et al. (2012, 2015, 2017), the handing over of such material was refused. Cryo-stocking of all strains and fruiting induction, with some modifications in single strains (Table 2), were carried out as previously described (Herzog et al. 2016). In each strain, fruiting was induced only when its vegetative mycelium had fully colonized the agar. Each fruiting experiment was repeated at least three times independently, each comprising three replicates.

**Table 1:** Strains sequenced and assessed for their fruiting characteristics in this study

Strain	Origin	Source	Reference	ITS	LSU	*TEF1α*	*RPB2*
*Cyclocybe aegerita* AAE-3	Parent strain *C. aegerita* 4022 reportedly isolated from *Buxus sempervirens* L. in Italy, 1970s (C. Chevalier, personal communication)	Sylvan Inc. (Horst, Netherlands)	Herzog et al. (2016), Gupta et al. (2018), Tayyrov et al. (2019)	MN306174	MN306154	MN308273	MN308254
*C. aegerita* CBS 127.88	Netherlands	CBS^a^	This study	MN306175	MN306172	MN308275	MN308255
*Cyclocybe* sp. (“*C. aegerita sensu* *lato”*; *Agrocybe chaxingu* Huang) SC960903	Thailand	IHI Zittau^b^	Gonzalez and Labarère (1998)	MN306176	MN306155	MN308276	MN308256
*C. aegerita* CBS 358.51	Italy	CBS^a^	This study	MN306177	MN306156	MN308277	MN308257
*C. aegerita* CBS 178.69	England	CBS^a^	This study	MN306178	MN306157	MN308278	MN308258
*Cyclocybe* sp. (“*C. aegerita* s.l.”) DSM 22459	GDR (East Germany), Jena-Winzerla, straw clamp, 1970, leg. G. Gramss	IHI Zittau^b^; DSMZ^c^	Ullrich et al. (2004)	MN306179	MN306158	MN308281	MN308261
*Cyclocybe* sp. (“*C. aegerita* s.l.”; *A. chaxingu* Huang) MES02023	China, Jilin Province	WUR^d^	This study	MN306180	MN306159	MN308283	MN308263
*C. erebia* IHI606	Germany (DE), Lückendorf; Kurpark, 10/2017, leg. Dr. R. Ullrich	IHI Zittau^b^	This study	MN306181	MN306160	MN308279	MN308259
*C. aegerita* IHI8 (*C. aegerita* TM ae)	DE, Jena; isolated from deadwood	IHI Zittau^b^	This study	MN306182	MN306161	MN308280	MN308260
*C. aegerita* DSM 9613	Italy, leg. Dr. F. Zadrazil	DSMZ^c^	This study	MN306183	MN306162	MN308266	MN308247
*Cyclocybe* sp. (“*C. aegerita* s.l.”) IHI392	India, Indian state of Himachal Pradesh, leg. Dr. Ramesh C. Upadhyay	IHI Zittau^b^	This study	MN306184	MN306163	MN308267	MN308248
*C. aegerita* AaM	USA, Wisconsin, Madison, supermarket, CBSWy-241, leg. M. Kinne	IHI Zittau^b^	This study	MN306185	MN306164	MN308268	MN308249
*C. aegerita* IHI536	Italy, Bologna, supermarket, 10/2012, isolated by Dr. C. Liers	IHI Zittau^b^	This study	MN306186	MN306165	MN308269	MN308250
*Cyclocybe* sp. (“*C. aegerita* s.l.”; *A. chaxingu* Huang) IHI15	China, from a mushroom grower	IHI Zittau^b^	This study	MN306187	MN306166	MN308282	MN308262
*C. aegerita* CBS 832.87	unknown origin	CBS^a^	This study	MN306188	MN306167	MN308270	MN308251
*C. parasitica* ICMP 11668	New Zealand (NZ), Christchurch, isolated from *Plagianthus* sp.	ICMP^e^	This study	MN306189	MN306168	MN308271	MN308252
*C. parasitica* ICMP 16333	NZ, Ngaruawahia,? *Podocarpus* sp. (stump), 1995, leg. P. K. Buchanan	ICMP^e^	This study	MN306190	MN306169	MN308272	MN308253
*Agrocybe arvalis* DSM 9710	DE	DSMZ^c^	This study	MN306191	MN306170	MN308284	MN308265
*A. firma* CBS 390.79	unknown origin	CBS^a^	This study	MN306192	MN306171	MN308274	MN308264

^a^Westerdijk Institute (Utrecht, Netherlands, NL)

^b^International Institute (Zittau, Germany, DE)

^c^German Collection of Microorganisms & Cell Cultures (Braunschweig, DE)

^d^Wageningen University & Research (Wageningen, NL)

^e^International Collection of Microorganisms from Plants (Auckland, New Zealand, NZ)

**Table 2:** Modified fruiting setups in some strains of “*Cyclocybe aegerita* *sensu lato*”

Strain	Pre-induction temperature (°C)	Fruiting induction temperature (°C)
*C. aegerita* AAE-3	25	20
	30	26
*Cyclocybe* sp. (“*C. aegerita* s.l.”) SC960903	25	20
	30	26
	22	26
*Cyclocybe* sp. (“*C. aegerita* s.l.”) MES02023	25	20
	30	26
	22	26

For statistical assessment of potential differences in their basidiome sizes, mean values of cap diameter and stipe length were calculated based on 30 basidiomes of the European strain *C. aegerita* AAE-3 versus 30 basidiomes of *Cyclocybe* sp. IHI392 from India. A two-sample Student *t*- test was then used with the programme STATA/MP version 13.1 (StataCorp LLC, College Station, TX, USA) infer about the presence of statistically significant differences between the mean values of cap diameter and stipe length of both strains.

To examine whether an individual strain produced its basidiomes either randomly distributed or at defined spots on the cultivation medium surface, basidiomes produced by each strain were catalogued based on the position where they emerged. Positions were assigned in relation to one half of the surface of a 1.5% MEA, 90 mm-diameter Petri dish that was subdivided into four different zones starting from the centre: “centre”, “periphery”, and “edge”. The fourth zone is referred to as “point of injury” and circumvents a 0.5 cm^2^ hole in the periphery zone which was punched-out from the vegetative mycelium using a sterilized cork borer. A schematic representation of the zones is given in Fig. S1.

### Nuclear state verification

To verify each strain’s dikaryotic state, micro-cultivation chambers were assembled as described by Herzog et al. (2016). For each strain, a 2% MEA agar plug of 0.5 cm^2^ diameter overgrown by mycelium was inoculated on top of a glass slide of each chamber and covered with a microscope coverslip. Inoculated micro-cultivation chambers were incubated at 25 °C in the dark until at least 1 cm of hyphal outgrowth became visible (5–10 days depending on the strain). The dikaryotic state was verified by the presence of clamp connections between hyphal segments of each strain.

### Comparative assessment of basidiospore dimensions

Spore prints from mature basidiomes of selected strains were prepared as described for *C. aegerita* AAE-3 in Herzog et al. (2016). The mature basidiomes of these strains were grown in the axenic fruiting setup of Herzog et al. (2016), except for *Cyclocybe* sp. DSM 22459 which did not fruit in this fruiting setup. To yield spore prints of this strain, basidiomes production from spawn culture was applied. For that, a pre-culture plate was prepared first by centrally inoculating a 1.5% MEA plate followed by incubation at 25 °C in the dark for 14 days. The fully colonized plate was then chopped into pieces with a sterile scalpel and macerated for 15 s at maximum speed in 90 mL sterilized tap water using a T 25 digital Ultra-Turrax® handheld homogenizer (IKA, Staufen, Germany) mounted with an autoclaved disposable plastic dispersing element (S 25 D-14 G-KS, IKA). A 1 mL-aliquot of the homogenized mycelium was then transferred into each of four 250 mL Erlenmeyer flasks filled with 50 mL 2% malt extract liquid medium supplemented with 2% corn meal (Alnatura Produktions- und Handels-GmbH, Bickenbach, Germany) and grown for three weeks at 160 rpm on an orbital shaker at 24 °C. All four pre-cultures were poured into a mushroom spawn bag. The autoclaved spawn medium consisted of 200 g wheat straw supplemented with 20 g corn meal and 800 mL dH_2_O in an autoclave bag. Colonization of the spawn bag took place in darkness at room temperature. Fruiting was induced at room temperature over three months. For this, the colonized bag was cut open and placed into a wet chamber that was prepared analogously to those employed by Herzog et al. (2016). Spores were collected using petri dishes placed underneath the maturating mushrooms.

A subsample of each spore print was resuspended in 20 μL sterile dH_2_O, subsequently transferred to a glass slide, and covered by a cover slip. For each strain, length and width of 50 basidiospores were microscopically measured using a light microscope (Axio Lab.A1 microscope, Carl Zeiss AG, Oberkochen, Germany) equipped with Moticam 3.0 MP digital camera with Motic Images Plus 2.0 software (Motic Deutschland GmbH, Wetzlar, Germany). Values of spore length and width were visualized by means of box-and-whiskers plots, and significant differences among strains were assessed with analysis of variance (ANOVA) followed by the Tukey’s honestly significant difference *post hoc* test, after visually confirming normality and homoscedasticity of the data. Statistical analyses were performed in R v3.6.1 (R Core Team 2019).

### Isolation of fungal DNA

Genomic DNA from mycelium of each strain was isolated applying the CTAB protocol of Gupta et al. (2018). DNA concentration was measured using the Qubit dsDNA HS Assay Kit (Life Technologies GmbH, Darmstadt, Germany) on a Qubit® Fluorometer (Invitrogen, Carlsbad, CA, USA), following the manufacturer’s instructions.

### Amplification and sequencing of phylogenetic markers

Partial genomic sequences of the internal transcribed spacer regions and the 5.8S subunit (ITS), the ribosomal RNA large subunit (LSU), the translation elongation factor 1-α gene (*TEF1*α), and the RNA polymerase II subunit gene (*RPB2*), were obtained for all strains. The ITS and LSU regions were jointly amplified using primers V9G (de Hoog and van den Ende 1998) and LR8 (Vilgalys unpublished: www.botany.duke.edu/fungi/mycolab) in reactions with 50–100 ng of DNA template, 2 mM MgCl_2_, 0.2 mM dNTPs, 0.5 μM of each primer, and 2 U of S7 Fusion High-Fidelity DNA Polymerase (Art.-Nr.: 332530S, Biozym Scientific GmbH, Hessisch Oldendorf, Germany). Thermal cyclings consisted of a denaturation step at 94 °C for 4 min, 35 cycles at 94 °C for 30 s, 55 °C for 90 s, and 72 °C for 45 s, and a final elongation step of 72 °C for 5 min. Amplicons of correct size in 1% agarose electrophoreses were cut out from the gel and purified using the Zymoclean kit (D4001, Zymo Research Europe GmbH, Freiburg, Germany) following the manufacturer’s instructions, and then Sanger sequenced bidirectionally with primers ITS1F/ITS4 (White et al. 1990; Gardes and Bruns 1993) for the ITS region, and LR0R/LR7 (Hopple Jr and Vilgalys 1994) for LSU. Primers for partial *TEF1*α and *RPB2* amplification were designed by accessing the genome sequence of *C. aegerita* AAE-3 (Gupta et al. 2018, www.thines-lab.senckenberg.de/agrocybe_genome). Primers were checked by Oligocalc (http://biotools.nubic.northwestern.edu/OligoCalc.html) to ensure the absence of secondary structures and annealing temperature variations by more than 2 °C. Resulting *TEF1*α and *RPB2* primer sequences are listed in Table S1. For *RPB2*, alternatively, primers 5F_Eur and 7CR_Eur (Houbraken et al. 2012) were used where needed. PCR reactions were performed as described previously, but using temperature cycles of 98 °C for 30 s, 35 cycles at 98 °C for 20 s, 63 °C for 20 s, and 72 °C for 40 s, and a final step of 72 °C for 5 min. PCR products were purified and Sanger sequenced as described above, using the same primers for amplification. All sequences obtained in this study are deposited in GenBank under accession numbers MN306154–MN308284 (see Table 1 and Table S2). In some cases where PCRs yielded multiple bands, amplicons were cloned using the StrataClone PCR Cloning Kit (Agilent Technologies, Santa Clara, USA) following the manufacturer’s instructions. In these cases, amplification and sequencing was performed with primers M13F (−20) and M13R (−24).

### Phylogenetic analysis

Two independent phylogenetic reconstructions were performed. The first one aimed at establishing the phylogenetic relationships of the strains with other related species based on ITS and LSU sequences, whereas the second one explored more in detail the relationships among the strains from Table 1, which were also compared for their basidiome formation-related features.

The first analysis included, in addition to the strains under study, a selection of other strains representing species of Agaricales closest to *C. aegerita*. The analysis was based only on ITS and LSU sequences due to the low number of strains in NCBI GenBank represented by all four loci. Reference strains were selected by searching with BLAST (Altschul et al. 1990) the best GenBank matches against the ITS and LSU sequences of our strains, and retaining those strains represented by both loci. Other strains were selected manually, even if only represented by one locus, based on their known affinity to *C. aegerita*. Additionally, sequences of strains *Cyclocybe* sp. MG21 (isolated from mushrooms acquired on a local market in Yunnan or Sichuan, China, according to Li et al. 2018) and *C. salicaceicola* YAASM0711 (isolated from *Salix cavaleriei* in Zhongdian, Yunnan, China according to Chen et al. 2012) were retrieved from their published genomes (GenBank bioproject numbers: PRJNA454572 and PRJNA253770, respectively) using BLAST searches. Sequences of *Schizophyllum commune* were used as outgroup. Details of all reference strains included in this analysis are provided in Table S2. A first set of analyses was performed individually for each locus, by aligning each dataset using MAFFT v7.271 (Katoh and Standley 2013) with the G-INS-i parameters, and then removing ambiguously aligned regions with Gblocks v0.91b (Castresana 2000). RAxML v8.0 (Stamatakis 2014) was then used to build Maximum Likelihood (ML) phylogenies based on the GTRGAMMA model and 1000 bootstrap replicates. Genealogical concordance between the ITS and LSU ML trees was assessed using the partition homogeneity test implemented in the package *ape* v5.3 (Paradis et al. 2004) of R v3.6.1. Because both topologies did not differ (Fig. S2), a multilocus ML tree was built with RAxML after concatenating the ITS and LSU alignments, using the same settings described above but allowing for different model parameter estimations for each locus. A complementary phylogeny was built based on Bayesian analysis with MrBayes 3.2.2 × 64 (Ronquist et al. 2012), using the GTRGAMMA model, two independent MCMC runs for 10 M generations sampling every 100th generation, and a burn-in of 30% of the sampled trees. Convergence of the runs was checked using TRACER v1.6 (Drummond and Rambaut 2007).

The second phylogenetic analysis, based on ITS, LSU, *TEF1*α and *RPB2* sequences, included only the ones of the strains in Table 1 plus those of three additional strains of which all four loci are available (see Table S2). After assessing genealogical concordance among all four sequence sets (Fig. S3), multilocus ML and Bayesian phylogenies were obtained as described above. All alignments (Online Resources 1–2) and trees have been deposited in TreeBASE (accession number S25303).

## Results

### Two-locus tree confirms separation of *Agrocybe* spp. and *Cyclocybe* spp.

**Fig. 1:**
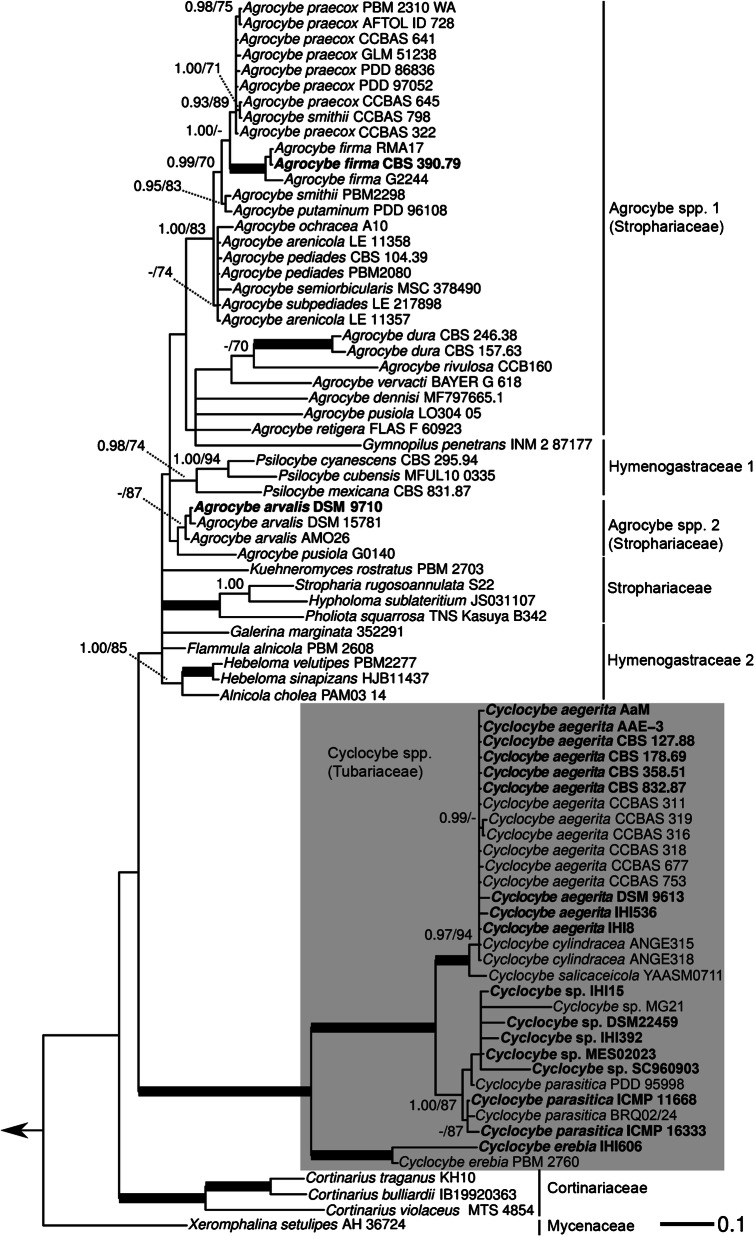
Maximum likelihood (ML) tree of *Agrocybe* spp. and *Cyclocybe* spp. towards a selection of hymenogastraceous or strophariaceous Agaricales taxa, based on a concatenated alignment of ITS and LSU sequences. Strains of *Agrocybe* spp. and *Cyclocybe* spp. also studied for their fruiting-related characteristics in this study are highlighted in bold. Support values above the branches: left side = % Bayesian inference posterior probability (PP); right side = % ML bootstrap value (BT) in absolute numbers. Branches of significant support (PP ≥ 0.99 and BT ≥ 95%) are thickened. Only support values of PP ≥ 0.90 and BT ≥ 70 are displayed for each node. Arrow in the left points to the outgroup (*Schizophyllum commune*)

The first phylogenetic reconstruction is based on ITS and LSU sequences and included, in addition to the strains under study, a selection of other strains representing agaric species, belonging to the families, according to He et al. (2019), Cortinariaceae, Hymenogastraceae, Mycenaceae, Schizophyllaceae (outgroup), and Strophariaceae (Fig. 1). This phylogeny shows a clear-cut separation between the genus *Agrocybe* (Strophariaceae), among others represented by several strains of *Agrocybe arvalis*, *Agrocybe dura*, *Agrocybe firma*, *Agrocybe pediades*, as well as *Agrocybe praecox*, and the genus *Cyclocybe* (Tubariaceae). *Agrocybe* species form two clusters of their own, although in a part of the phylogeny containing both members of the Hymenogastraceae and Strophariceae in unresolved relationship towards each other. There, together with all included *Agrocybe* spp., typical Strophariaceae like *Hypholoma sublateritium* or *Stropharia rugosoannulata* form a little supported cluster together with members of the Hymenogastraceae, such as three species from the genus *Psilocybe*, *Gymnopilus penetrans*, *Flammula alnicola*, *Hebeloma velutipes*, or *Galerina marginata*.

Within the genus *Cyclocybe*, two main clades are resolved, one comprising *C. erebia* and the other one “*C. aegerita sensu* *lato*”, *C. parasitica* and *C. salicaceicola*. Strains of “*C. aegerita* s.l.” split up, clustering across two separate but not fully resolved branches. The first one almost exclusively comprises strains of European origin, except for *C. aegerita* (AaM) that was isolated from basidiomes bought in a US supermarket. The Chinese species *C. salicaceicola* (solely based on *C. salicaceicola* YAASM0711 from Yunnan, China), a close relative of *C. aegerita* according to Yang et al. (1993) and Chen et al. (2012, 2015, 2017), groups in a well-supported sister clade towards the European lineage of “*C. aegerita* s.l.”. The second major branch of “*C. aegerita* s.l.” includes all its Asian strains, all assigned to *Cyclocybe* sp., and one outlier (*C. parasitica* PDD 95998) of the Pacific species *C. parasitica* from New Zealand. All other *C. parasitica* strains form a potential sister clade relationship to the outlier and *Cyclocybe* sp. which exclusively comprises Asian strains with the exception of *Cyclocybe* sp. DSM 22459 that was originally isolated from a straw pile in Jena (East Germany) in 1970. The phylogenetic relatedness of the strains within the Asian clade to each other and their relationship to *C. parasitica* is not sufficiently resolved within the two-locus phylogeny. The same is true for the European group of “*C. aegerita* s.l.” that also includes two strains of *C. cylindracea*.

### Multilocus tree-based division of “*C. aegerita* s.l.” into two diverging monophyla

**Fig. 2:**
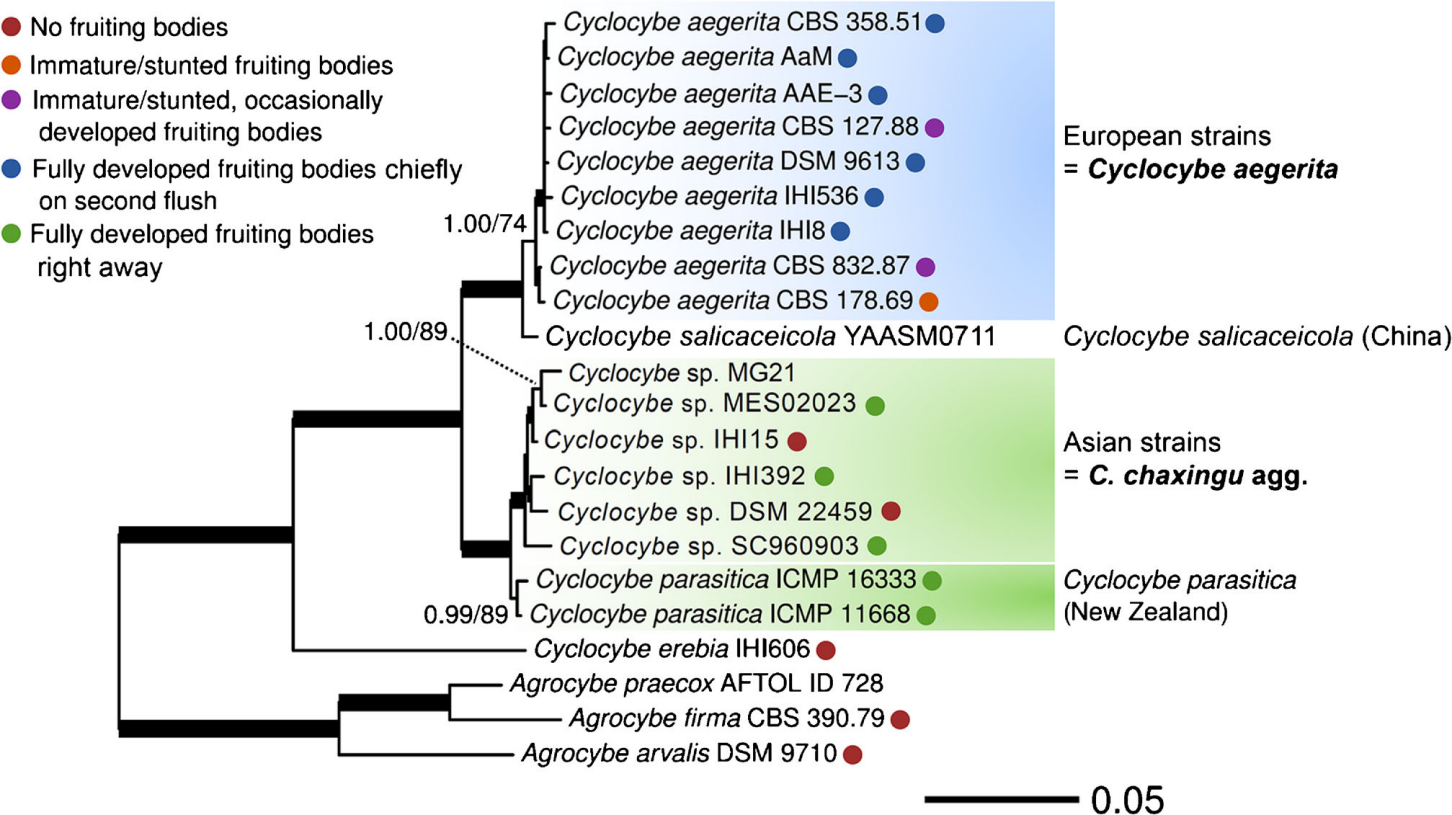
Maximum likelihood (ML) tree of *Cyclocybe aegerita* and adjacent species, based on a concatenated multigene alignment of ribosomal (ITS, LSU) and proteinogenic (*TEF1α*, *RPB2*) DNA sequences. Support values above the branches: left side = % Bayesian inference posterior probability (PP); right side = % ML bootstrap value (BT) in absolute numbers. Branches of significant support (PP ≥ 0.99 and BT ≥ 95%) are thickened. Only support values of PP ≥ 0.90 and BT ≥ 70 are displayed for each node. Coloured circles encode the fruiting performance of each tested strain in the fruiting setup of Herzog et al. (2016); the term 'stunted' means that either aborted immature basidiomes and/or aborted primordia were seen with a certain strain

The second phylogenetic reconstruction is based on four genetic markers, including ribosomal (ITS, LSU) and proteinogenic (*TEF1α* and *RPB2*) DNA sequences. Taxon sampling includes one strain each of *A. arvalis*, *A. firma*, and *A. praecox*, as well as several geographically distant strains of “*C. aegerita* s.l.” (including three strains of *A. chaxingu a priori* subsumed here, see Table 1) and strains of its close relatives *C. erebia*, *C. parasitica*, and *C. salicaceicola* (Fig. 2). Except for three strains (*C. salicaceicola* YAASM0711, *Cyclocybe* sp. MG21, and *A. praecox* AFTOL ID 728), the fruiting performance was determined experimentally for all strains employed in this analysis applying the fruiting setup of Herzog et al. (2016). This yielded different degrees of fruiting productivity as depicted by the coloured circles in Fig. 2.

In comparison to the tree in Fig. 1, that of Fig. 2 confirms major results of the former but at a significantly higher resolution. Thus, the clear separation of *Agrocybe* spp. from *Cyclocybe* spp. and the relationship of *C. erebia* as a sister clade of “*C. aegerita* s.l.”, *C. salicaceicola*, and *C. parasitica* of the two-locus phylogeny was confirmed and reappeared even more pronounced in the multilocus phylogeny. The multilocus tree fully resolves the splitting of “*C. aegerita* s.l.” into two monophyletic clades and sharply delimits them from their sister monophyla *C. salicaceicola* and *C. parasitica*. The first lineage of “*C. aegerita* s.l.” includes solely European strains (except for *C. aegerita* AaM). It clearly separates from its second lineage made up by strains from Asia (except for the East German straw heap isolate *Cyclocybe* sp. DSM 22459 that seems to represent a species of its own to be described in the future). We propose to delimit these Asian strains from the European *C. aegerita* as a clearly separate monophylum, preliminarily referred to as *Cyclocybe chaxingu* agg., which may comprise several species (species complex).

The latter scenario is supported by the subclade structure of the Asian monophylum. In a first subclade, the three strains from China, i.e. *Cyclocybe* sp. MG21, *Cyclocybe* sp. MES02023, and *Cyclocybe* sp. IHI15 cluster together, with the first two strains forming a sister clade of the latter one, and the latter two strains being referenced as *A. chaxingu* (see Table 1). The first subclade forms a sister group to *Cyclocybe* sp. IHI392 from India and *Cyclocybe* sp*.* DSM 22459 which make up a second subclade. The Thai strain *Cyclocybe* sp. SC960903 (another *A. chaxingu* strain after Gonzalez and Labarère 1998, see Table 1) forms an outgroup to the two former subclades.

With respect to their fruiting productivity, *C. aegerita* strains differed visibly from their Asian relatives and the Pacific species *C. parasitica*. In this context, “*C. aegerita* s.l.” and *C. parasitica* also differ from the strains of *C. erebia*, *A. arvalis*, and *A. firma* studied here (see respective colour code in Fig. 2).

### Fruiting features of *C. aegerita* versus its relatives from Asia and New Zealand

**Fig. 3:**
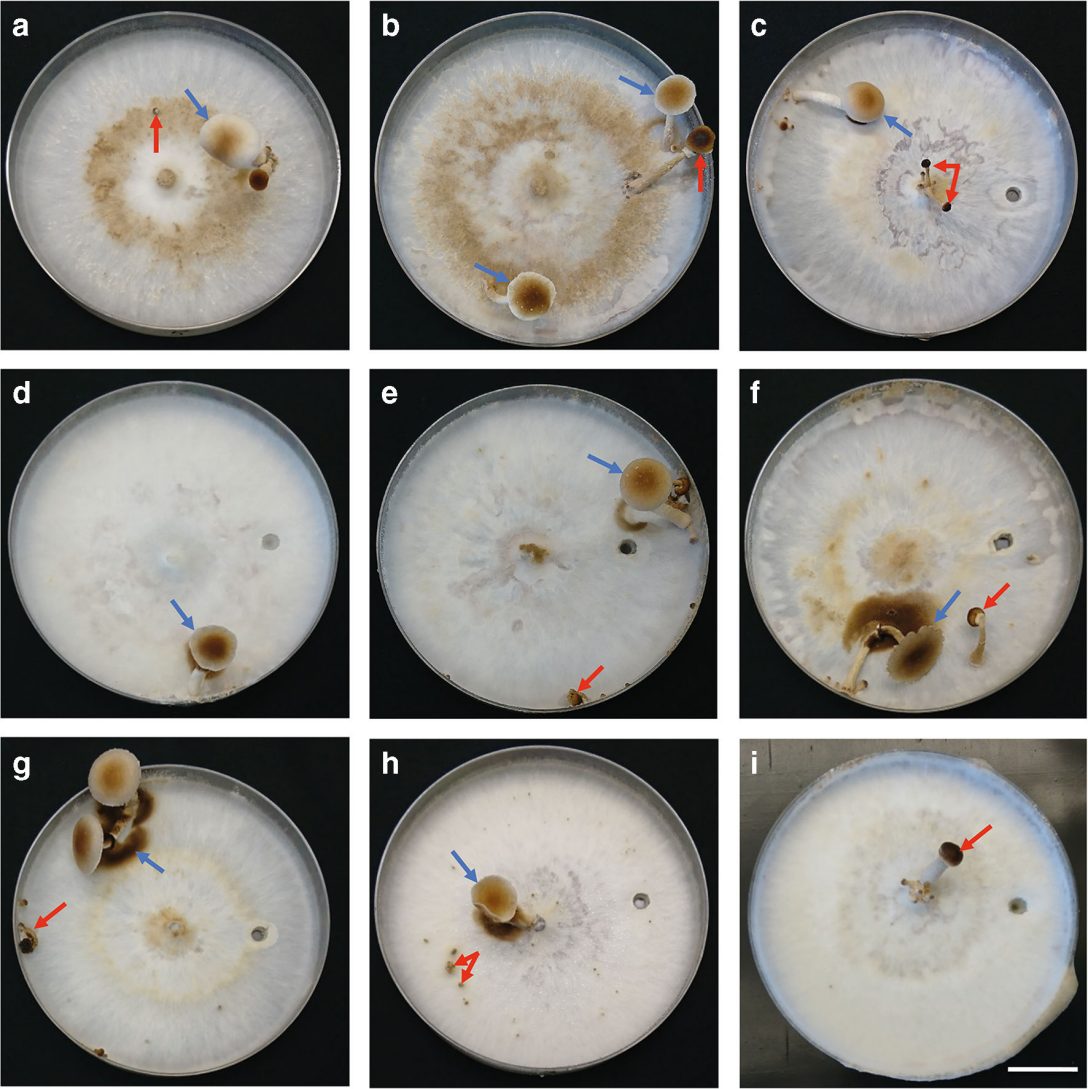
Fruiting characteristics of European *Cyclocybe aegerita* strains in the fruiting setup of Herzog et al. (2016), 25–50 days post-inoculation (pre-incubation, pi: 11–13 d at 25 °C in the dark; fruiting induction, fi: 15–39 d at 20 °C 12 h light/12 h dark). Blue arrows point to mature basidiomes (FBs), and, if not specified differently, red arrows point to stunted immature FBs. (**a**) Italian *C. aegerita* strain CBS 358.51, 13 d pi, 15 d fi; red arrow points to a stunted primordium. (**b**) Strain *C. aegerita* AaM isolated from *C. aegerita* mushrooms bought in a US supermarket, 12 d pi, 35 d fi. (**c**) Genome-sequenced strain *C. aegerita* AAE-3 derived from the reportedly Italian strain *C. aegerita* 4022, 11 d pi, 30 d fi. (**d**) Dutch strain *C. aegerita* CBS 127.88, 11 d pi, 39 d fi. (**e**) Italian strain *C. aegerita* DSM 9613, 12 d pi, 36 d fi. (**f**) Strain *C. aegerita* IHI536 isolated from *C. aegerita* mushrooms bought in an Italian supermarket, 11 d pi, 36 d fi. (**g**) German strain *C. aegerita* IHI8, 11 d pi, 36 d fi. (**h**) Strain *C. aegerita* CBS 832.87 of unknown origin, 13 d pi, 25 d fi; red arrows point to stunted primordia. (**i**) English strain *C. aegerita* CBS 178.69, 11 d pi, 18 d fi. Bar = 2 cm

**Fig. 4:**
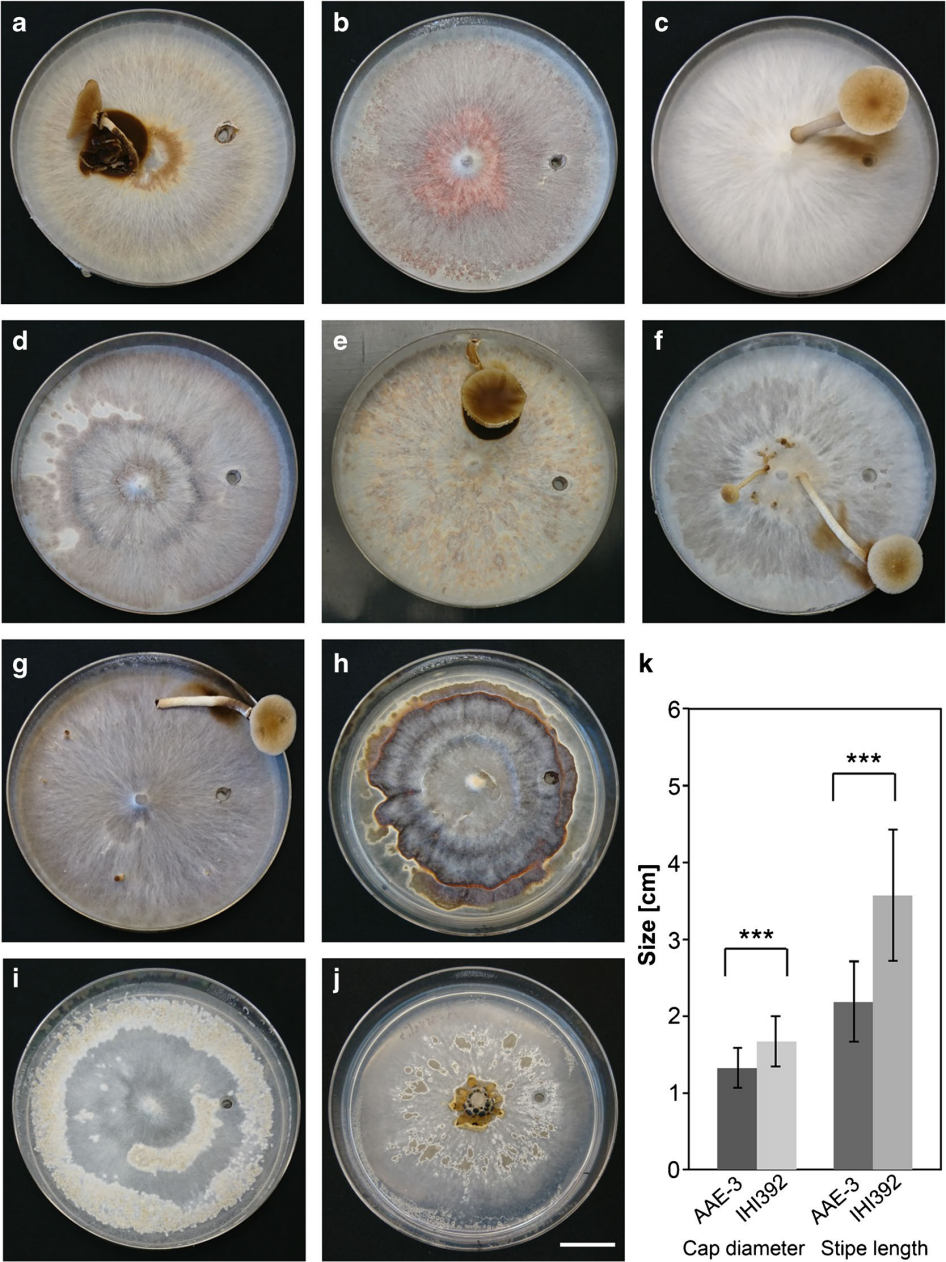
Fruiting features of (**a**)–(**j**) Strains from the Asian monophylum/monophyletic species complex preliminarily named *Cyclocybe chaxingu* agg., *C. erebia*, *Agrocybe firma* and *A. arvalis*, and statistic evaluation of basidiome dimensions of (**k**) a *C. aegerita* strain versus a strain from the Asian monophylum in the fruiting setup of Herzog et al. (2016), 25–70 days post-inoculation (pre-incubation, pi: 12–37 d at 25 °C in the dark; fruiting induction, fi: 12–41 d at 20 °C 12 h light/12 h dark), with modifications where specified. (**a**) Chinese strain *Cyclocybe* sp. MES02023 displaying a mature basidiome (FB), 12 d pi, 33 d fi. (**b**) Chinese breeding strain *Cyclocybe* sp. IHI15 only showing mycelium, 13 d pi, 37 d fi. (**c**) Indian strain *Cyclocybe* sp. IHI392 exhibiting a mature FB, 13 d pi; 12 d fi. (**d**) East German strain *Cyclocybe* sp. DSM 22459 only displaying mycelium, 19 d pi, 35 d fi. (**e**) Thai strain *Cyclocybe* sp. SC960903 exhibiting a mature FB 14 d pi at 30 °C, 41 d fi at 26 °C. (**f**) New Zealand strain *C. parasitica* ICMP 16333 displaying a mature FB, 15 d pi, 35 d fi. (**g**) New Zealand strain *C. parasitica* ICMP 11668 showing a mature FB, 13 d pi, 21 d fi. (**h**) German strain C. *erebia* IHI606 exhibiting mycelium with brown pigmentation, 22 d pi, 35 d fi. (**i**) Strain *A. firma* CBS 390.79 (unknown origin) showing initial fruiting stages, 29 d pi, 34 d fi. (**j**) German strain *A. arvalis* DSM 9710 displaying mycelium, 37 d pi, 33 d fi. Bar = 2 cm. (**k**) Statistical comparison by student’s t test of mean cap diameter and mean stipe length of FBs from *C. aegerita* AAE-3 versus *Cyclocybe* sp. IHI392. Error bars represent the standard deviation (*n* = 30). *** indicates a *P* < 0.001

Applying the fruiting setup by Herzog et al. (2016) with small modifications for a few individual strains (see Table 2), mature basidiomes were ultimately formed by thirteen out of nineteen tested strains from the genera *Cyclocybe* and *Agrocybe*, and immature basidiomes were formed by one strain (Figs. 3 and 4). All strains were confirmed to be dikaryotic (Fig. S4) and could be assigned to subgroups based on their fruiting productivity ranging from abundant production of mature basiomes to no fruiting at all (see Fig. 2, Figs. 3–4, and Figs. S5–S8). A more variable spectrum of fruiting productivities was recorded among strains of *C. aegerita* from Europe, ranging from highly productive to fairly productive strains. This is in contrast to its relatives from Asia and New Zealand, in the case of which individual strains either exhibited no fruiting at all or an even more efficient production of mature mushrooms than all European strains, i.e. an almost exclusive immediate production of mature basidiomes in the first fruiting flush (see Figs. 2–4, Figs. S5–S8).

Within their first fruiting flush, the fruiting-wise most productive subgroup within *C. aegerita*, comprising *C. aegerita* CBS 358.51, *C. aegerita* AAE-3, *C. aegerita* DSM 9613, *C. aegerita* AaM, *C. aegerita* IHI536, and *C. aegerita* IHI8, mostly but not exclusively produced basidiomes remaining in the stage of immaturity, e.g. lacking full cap expansion and spore shedding. However, they also produced several mature mushrooms alongside the immature ones, exemplarily shown by *C. aegerita* CBS 358.51 (Fig. 3a) or *C. aegerita* IHI536 (Fig. S6b, right photo). In their second fruiting flush, those strains mainly produced fully developed mushrooms (Fig. 3b, c, e–g). The second subgroup, made up by *C. aegerita* CBS 127.88 and *C. aegerita* CBS 832.87, mainly formed stunted immature basidiomes, i.e. mature basidiomes were only occasionally observed (Fig. 3d, h, Fig. S5d, and Fig. S6d). The third subgroup consists solely of *C. aegerita* CBS 178.69, which only produced immature mushrooms (Fig. 3i and Fig. S7a).

Among strains from the newly delimited Asian species complex, and the Pacific species *C. parasitica*, two fruiting productivity subgroups were categorized. Most strains of the former, except for *Cyclocybe* sp. IHI15 and *Cyclocybe* sp. DSM 22459, and all *C. parasitica* strains, produced almost only mature basidiomes already in their first fruiting flush (Fig. 4a, c, e–g, Fig. S7b–d, and Fig. S8). The mentioned exceptions (Fig. 4b, d) did not fruit within the fruiting setup of Herzog et al. (2016). Similarly, the fruiting-induced strains *C. erebia* IHI606, *A. firma* CBS 390.79, and *A. arvalis* DSM 9710 also did not fruit under these conditions (Fig. 4h–j). Still, they either produced some brownish pigments instead, with an extensive brown pigmentation in the case of *C. erebia* IHI606 and a more scattered light brown one in *A. arvalis* DSM 9710 (Fig. 4h, j), or they at least showed initial signs of fruiting as observed for *A. firma* CBS 390.79 (Fig. 4i).

Qualitatively, two additional aspects of the general fruiting patterns among the newly delimited Asian monophylum and *C. parasitica* versus *C. aegerita* were noticed. On the one hand, we recorded that the overall amount of basidiomes produced by the strains of *C. aegerita* was generally higher than the amount produced by the Asian strains (Table S3). On the other hand, in return, representatives of the proposed Asian monophylum and *C. parasitica* seem to form bigger basidiomes compared to the ones produced by *C. aegerita* (see Figs. 3–4 and Figs. S5–S8). We checked this visual assessment by a statistical comparison of the stipe length and the cap diameter of mushrooms either harvested from the European strain *C. aegerita* AAE-3 or from the Asian strain *Cyclocybe* sp. IHI392. Statistic evaluation of the results shows that the Asian strain produces mushrooms of significantly longer stipe length and significantly wider cap diameter (Fig. 4k).

### Basidiospore size in *C. aegerita* versus its relatives from Asia and New Zealand

**Fig. 5:**
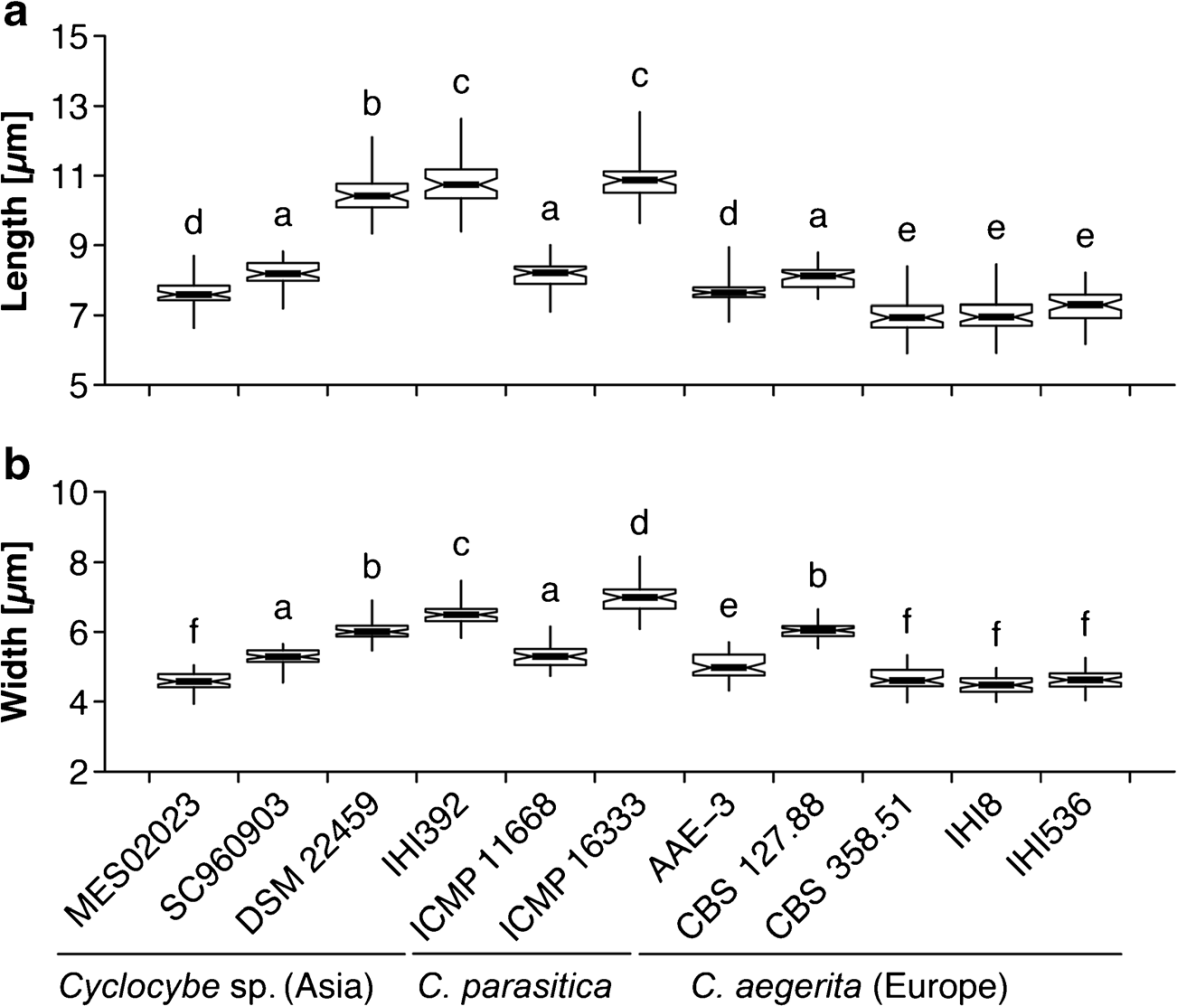
Box-and-whisker plots showing the distribution of spore length (**a**) and spore width (**b**) values across the studied strains of “*Cyclocybe aegerita sensu lato*”, and *Cyclocybe parasitica*.. Different letters above boxes indicate differences at *P* < 0.05 as assessed by the Tukey’s honestly significant difference *post hoc* test

From the assessed strains of *C. aegerita*, the Asian species complex we delimited from our multilocus phylogenetic analysis, and *C. parasitica*, *Cyclocybe* sp. IHI392 and *C. parasitica* ICMP 16333 display the longest and the widest spores of all tested strains (Fig. 5). The European strains *C. aegerita* CBS 358.51, *C. aegerita* IHI8, and *C. aegerita* IHI536 exhibit both the shortest spores (Fig. 5a) and the slimmest spores, the latter together with *Cyclocybe* sp. MES02023 from China (Fig. 5b).

Although the shortest spores were seen among *C. aegerita* strains (CBS 358.51, IHI8, and IHI536) and the longest ones among the second subclade of the Asian monophylum (IHI392, and the geographic outlier DSM 22459; see Fig. 2), there were also two Asian strains (SC960903, and MES02023) with spores about as short as in the remaining two *C. aegerita* strains (CBS 127.88, and AAE-3). A comparable variability applies between the two strains of *C. parasitica*, one of which (ICMP 11668) shows a spore length as in the longest-spored European strain (CBS 127.88), while the spores of the other one are as long as those of *Cyclocybe* sp. IHI392 (see Fig. 5a). In the case of the spore width, we also recorded a considerable strain-specific variability within *C. aegerita*, across strains from the newly delimited Asian monophylum, as well as across the two strains of *C. parasitica* (see Fig. 5b).

### Altered fruiting temperature in individual Asian strains and fruiting patterns

In the strain *Cyclocybe* sp. SC960903, in contrast to the control strains *C. aegerita* AAE-3 (Fig. S9a) and *Cyclocybe* sp. MES02023 (Fig. S9b), no fruiting was achieved when it (Fig. S9c) faced the default cultivation and fruiting regime by Herzog et al. (2016). However, increasing the temperature to 30 °C during vegetative growth and to 26 °C for fruiting induction, ultimately, in contrast to both control strains (Fig. S9d, e), yielded fruiting in *Cyclocybe* sp. SC960903 (Fig. S9f). A third temperature regime comprising a temperature of 22 °C during vegetative growth and one of 26 °C for fruiting induction only lead to fruiting in the control strain *Cyclocybe* sp. MES02023 (Fig. S9g, h).

Most strains seemingly produced their basidiomes randomly distributed over the surface of the cultivation medium. Still, some of them exhibited a pattern where they preferentially produce them (see Fig. S1, Table S3, and Figs. S5–S8). The Italian strain *C. aegerita* CBS 358.51 nearly exclusively fruited at the point of injury, where the mycelium was injured by punching out a 0.5 cm^2^ agar plug to locally stimulate fruiting (see Fig. 3a, Fig. S5a, and Table S3). Furthermore, this strain produced high numbers of basidiome initials, with only a few of them developing into mushrooms. The only other strain that preferentially fruited at the point of injury, but by far not as exclusively as *C. aegerita* CBS 358.51, was *C. aegerita* AaM (see Fig. 3b, Fig. S5b, and Table S3). *Cyclocybe aegerita* CBS 178.69 exhibited a strong preference to fruit in the plate centre, however, only producing immature mushrooms that often emerged directly on the inoculation plug of the plate (see Fig. 3i, Fig. S7a, and Table S3). Similarly, *C. aegerita* CBS 832.87 preferentially fruited in the plate centre, also especially on the inoculation plug (see Fig. 3h, Fig. S6d, and Table S3). In contrast to them, *C. aegerita* IHI8 mostly avoided fruiting in or nearby the centre as it almost exclusively fruited at the plate edge (see Fig. 3g, Fig. S6c, and Table S3). *Cyclocybe parasitica* ICMP 11668 showed a similar fruiting pattern as *C. aegerita* IHI8 but preferred not to fruit “far away” from the centre, by chiefly fruiting in the peripheral zone (see Fig. 4g, Fig. S8b, and Table S3).

A few general positional fruiting preferences can be noticed for *C. parasitica* and the newly delimited Asian monophylum versus *C. aegerita*: The former species do not fruit at the point of injury at all. On average, *C. aegerita* strains most abundantly fruited in the zone around the plate centre, while their Asian relatives showed an about fifty-fifty divided fruited preference between plate edge and plate centre. In contrast, *C. parasitica* most frequently fruited in the peripheral zone (Table S3).

## Discussion

### Multilocus phylogeny of *Cyclocybe* spp. harmonizes with their fruiting features

Our two-locus-based phylogeny of concatenated ITS and LSU sequences confirms the single locus-based findings by Vizzini et al. (2014), who, thus, assigned *Agrocybe* spp. and *Cyclocybe* spp. to separate Agaricales families. These assignments were recently updated by He et al. (2019). Accordingly, our results add further support to the resurrection of the genus *Cyclocybe* for *C. aegerita* (V. Brig.) Vizzini, *A. chaxingu* Huang (Zhi 1991) in this study *a priori* assigned to “*C. aegerita* s.l.”, *C. parasitica* (G. Stev.) Vizzini, *C. salicaceicola* (Zhu L. Yang, M. Zang & X.X. Liu) Vizzini, and *C. erebia* (Fr.) Vizzini & Matheny. Due to our focus on “*C. aegerita* s.l.”, a check-up on *C. erebioides* Angelini & Vizzini, which clusters between *C. erebia* and “*C. aegerita* s.l.” in the single locus trees of Vizzini et al. (2014), was not pursued in this study.

We are also aware of the fact that the species status of *C. aegerita* towards *C. cylindracea* is not completely clear. Currently, both names are valid according to Index Fungorum. Nevertheless, the focus of the present study was not to clarify whether both species are potentially conspecific based on type specimens. We chiefly aimed at elucidating the status of the Asian species complex of “*C. aegerita* *sensu* *lato*” and the Pacific species *C. parasitica* towards strains from Europe. Thus, even though our two-locus tree contains ITS + LSU sequences from two non-type specimens assigned to *C. cylindracea*, which cluster among European *C. aegerita* (see Fig. 1), we refrain from suggesting that both taxa may be conspecific. Such would entail suggesting a bold nomenclatural change giving *C. cylindracea*, based on the older name *Agaricus cylindraceus* DC. 1815, if not a sanctioned name, priority over *C. aegerita* (based on *Agaricus aegerita* Brig. 1837). However, such a proposition must instead be based on solid groundwork, i.e. on unrelenting efforts to obtain molecular (for a multilocus analysis) and morphological data of type material or epitypes generated by sampling-intensive fieldwork from the *locus typi* of each species.

The same stipulations apply to the number of species to diagnose within the newly delimited Asian monophylum/potential species complex, we preliminarily refer to as *C. chaxingu* agg., and which our multilocus phylogeny allowed to separate from *C. aegerita*. The Pacific species *C. parasitica* revealed itself as a sister clade to this potential species complex which was also reflected by their similar fruiting properties (see Fig. 4, Fig. S7b–d, and Fig. S8). Moreover, the fact that spore size seems to vary across the subclades of the monophylum we preliminarily refer to as *C. chaxingu* agg. (see Fig. 2 and Fig. 5) provides additional reason to expect it getting further characterized as a species complex in future studies with larger taxon samplings including basidiomes and spore prints from specimens collected in the field which should include the *locus typi* of the name giving species.

The multilocus tree also confirmed the tight association of *C. salicaceicola* to *C. aegerita*. This is an interesting finding as *C. salicaceicola* was originally described from Yunnan (China) as a species that is morphologically similar to *C. aegerita* according to Yang et al. (1993) and hardly to distinguish from *C. aegerita* according to Chen et al. (2012), who based their conclusions on molecular species differentiation. Unfortunately, our official request for strains of *C. salicaceicola* to the authors of Chen et al. (2012, 2015, 2017) was refused with the statement that it is their core resource, which they cannot give abroad. Hence, we could not check whether the fruiting properties of *C. salicaceicola* may resemble those of *C. aegerita*. This would have helped further reflecting upon the taxonomic relations between these species. Nevertheless, it is still a remarkable finding that *C. salicaceicola* YAASM0711, despite its geographic origin, clusters more closely to *C. aegerita* strains from Europe. Our finding is still based on just one four-locus dataset from one *C. salicaceicola* strain. Therefore, it should be reassessed by future multilocus-based phylogenetic analyses including a set of *C. salicaceicola* strains.

Within each of the two clades of “*C. aegerita* s.l.”, we noticed two geographic outliers. Within the *C. aegerita* clade, only *C. aegerita* AaM originates from outside Europe as it was isolated from commercially acquired basidiomes from a supermarket in Madison, Wisconsin, USA. The status of *C. aegerita* as a cultivated edible mushroom in numerous countries worldwide (Uhart et al. 2008) may provide one hypothetic explanation for this finding. Potentially, an originally European production strain may have been sold to mushroom growers/vendors in North America, or edible mushrooms originally produced in Italy, the second biggest player in this industry (Friedman 2016). Our observation on the common individual fruiting pattern exhibited by both the US strain *C. aegerita* AaM and the Italian strain *C. aegerita* CBS 358.51 to fruit preferentially upon injury stimuli may, thus, not just be coincidence. Prospective comparative genomics or transcriptomics studies versus a strain, like the genome-sequenced one *C. aegerita* AAE-3 (Gupta et al. 2018), that seemingly never fruits where its mycelium is injured, may detect putative genetic alterations or expression patterns of fruiting-related genes in *C. aegerita* CBS 358.51 and *C. aegerita* AaM accounting for their specific fruiting pattern.

Within the newly delimited Asian monophylum, the East German strain *Cyclocybe* sp. DSM 22459 is an odd geographic outlier. It was isolated in 1970 from a straw pile in the outskirts of Jena (Thuringia, Germany) by Gerhard Gramss, a renowned mycologist who has been actively publishing on basidiomycetous fungi over five decades (Gramss 1979, 1980; Gramss et al. 1999; Gramss and Bergmann 2008; Gramss and Voigt 2013). To see whether this strain will remain the only geographic ‘oddball’ within the Asian monophylum, it will certainly help to extend the taxon sampling in the region around Jena and in similar places in Thuringia to obtain more specimens of *Cyclocybe* spp./“*C. aegerita* s.l.” One still may speculate that the strain *Cyclocybe* sp. DSM 22459 came to Jena in 1950s or 1960s in the course of academic collaborations of the Jena university and other research institutions with partners in China and Vietnam. Since this strain is the prototypical producer of wild-type peroxygenase (UPO, EC 1.11.2.1) – a hotspot of current biocatalytic research (Wang et al. 2017) – future studies comparing the peroxygenase levels and isoenzyme patterns of different *Agrocybe/Cyclocybe* spp. will help as well to further disentangle its position within the respective phylogenetic tree (Ullrich et al. 2004; Hofrichter et al. 2015, 2020). In this context, the description of this strain as a species of its own appears to be plausible.

### Metabolism-related aspects of fruiting features from different *Cyclocybe* spp.

Some strains did not fruit at all within the default fruiting setup of Herzog et al. (2016). In *Cyclocybe* sp. SC960903, this could be changed by applying a different temperature regime within the cultivation setup of Herzog et al. (2016), i.e. a higher vegetative growth and fruiting induction temperature (see Fig. S9f). Given the Southeastern Asian origin of this strain, an ecotype-like adaptation of this strain can be assumed, linking the induction of basidiome formation with environmental cues related to the tropical monsoon climate in Thailand. This conclusion is supported by the failure of *Cyclocybe* sp. MES02023 to fruit in the 30 °C/26 °C regime (see Fig. S9e). This strain originates from the Jilin Province in Northern China, which is characterized by temperate climate. The European reference strain *C. aegerita* AAE-3 (Herzog et al. 2016; Gupta et al. 2018) also failed to fruit at elevated temperature (see Fig. S9d), which may indicate that its parent strain (see Table 1) also rather originates from a region in Italy that is characterized by moderately warm climate.

In other cases, where no fruiting was achieved by applying the default fruiting setup of Herzog et al. (2016), for instance, in *Cyclocybe* sp. DSM 22459, fruiting could be achieved by using a mushroom spawn substrate instead (see Material and Methods). On the one hand, such ‘behaviour’ may relate to physiological requirements of the particular strain, which can simply exceed the nutrient amounts required for fruiting from an agar plate. According to Chanter (1979), nutrition should accumulate as a ‘storage substrate’ in the mycelium and fruiting is initiated only when the substrate density in the mycelium exceeds a threshold level. On the other hand, the phenomenon that fructification cannot be induced on agar plates is common in other commercially grown mushroom species such as *Agaricus bisporus* or *Lentinula edodes,* where only a complex voluminous mushroom spawn substrate allows fruiting, being either compost with a casing layer (Morin et al. 2012; Straatsma et al. 2013) or a nearly exclusively wood-based substrate (Chen et al. 2016).

Testing the other non-fruiters from the present study, i.e. *Cyclocybe* sp. IHI15, *C. erebia* IHI606, *A. firma* CBS 390.79, or *A. arvalis* DSM 9710 for their fruiting capability on a spawn substrate as applied in *Cyclocybe* sp. DSM 22459, and, if applicable, also within a customized temperature regime, might eventually lead to basidiome production under laboratory conditions with these strains.

### Implications of the biogeographic diversity for *C. aegerita* as a model organism

*C. aegerita* is used as model system to study or exploit diverse capabilities of mushroom-forming basidiomycetous fungi including fruiting (Herzog et al. 2016), the production of biotechnologically relevant enzymes (Hofrichter et al. 2020) or the biosynthesis of various metabolites including volatiles (Zhao et al. 2003; Ngai et al. 2005; Kögl et al. 2007; Kleofas et al. 2014; Hennicke et al. 2019; Surup et al. 2019; Tayyrov et al. 2019; Orban et al. 2020). Therefore, the here reported split-up of “*C. aegerita* s.l.” into a European and an Asian monophylum/species complex brings along some practical implications for these research fields. So far, approaches with an interest towards gene functions were carried out with the genome-sequenced (Gupta et al. 2018) European strain *C. aegerita* AAE-3 (Herzog et al. 2019; Surup et al. 2019; Tayyrov et al. 2019). By sequencing genomes of strains from the Asian monophylum/species complex, preliminarily named *C. chaxingu* agg. (including *Cyclocybe* sp. DSM 22459), one can expect to find new genes/alleles encoding, e.g., fruiting-related proteins. Also, this may reveal new variants of ribotoxins, terpenoids, peroxygenases, peroxidases or other carbohydrate active enzymes differing from those of *C. aegerita* AAE-3 (Gupta et al. 2018; Surup et al. 2019; Tayyrov et al. 2019). Such data will be of general interest to a broad scientific community dealing with natural products chemistry including volatiles (Kleofas et al. 2014; Citores et al. 2019; Surup et al. 2019; Tayyrov et al. 2019; Orban et al. 2020), enzyme biochemistry and biotechnology (Hofrichter et al. 2015; Wang et al. 2017; Karrer and Rühl 2019; Hofrichter et al. 2020), or developmental biology (Herzog et al. 2016).

## Conclusion

The present study indicates a well-supported delimitation of a new Asian species complex from “classic” *C. aegerita*, a result that is supported by the fruiting properties of respective strains. Furthermore, a sister group affiliation of this species complex to *C. parasitica* and of *C. aegerita* to *C. salicaceicola* has been elucidated. Given that fruiting properties differ between *C. aegerita* versus its relatives from Asia and New Zealand, as well as between certain individual strains, we can speculate in how far they emerge as a result of selective pressure, potentially manifesting as ecotype-like adaptations. Future comparative genomics analyses will help to unravel how genetic differences may have translated into differing fruiting properties. Such knowledge will also extend our understanding of the origin and function of biodiversity in basidiomycetous mushrooms from genes to ecotypes based on genomic diversity.

## Electronic supplementary material

Fig. S1 Subdivision of the cultivation mediums surface into different zones. The edge zone (I) spans 1.5 cm starting from the plate edge and ending at the distal end of the periphery zone (II). The periphery zone measures 2.25 cm from the end of the edge zone edge towards the plate centre, ending at the distal end of the centre zone. The centre zone (III) covers 0.75 cm from the plate centre towards the periphery zone. The point-of-injury zone (IV) is made up by a 0.5 cm^2^-punched-out hole in the distal area of the periphery zone (PNG 244 kb)

High Resolution (TIF 282 kb)

Fig. S2 Maximum likelihood (ML) trees of *Agrocybe* spp. and *Cyclocybe* spp. towards a selection of hymenogastraceous or strophariaceous Agaricales taxa, based on a ITS or LSU sequences. Support values above the branches: ML bootstrap value (BT) in absolute numbers. Only support values of BT ≥ 70 are displayed for each node (PNG 1018 kb)

High Resolution (TIF 1232 kb)

Fig. S3 Maximum likelihood (ML) trees of *Agrocybe* spp. and *Cyclocybe* spp., based on ITS, LSU, *RPB2*, or *TEF1α* sequences. Support values above the branches: ML bootstrap value (BT) in absolute numbers. Only support values of BT ≥ 70 are displayed for each node (PNG 629 kb)

High Resolution (TIF 966 kb)

Fig. S4 Clamp formation by different dikaryotic strains of *Cyclocybe aegerita*, from the Asian monophylum/monophyletic species complex preliminarily named *C. chaxingu* agg., and from *C. parasitica*, if not specified differently, grown in a micro-cultivation chamber for 7 days at 25 °C. If not specified differently, bar = 20 μm. White arrows mark the position of clamp connections in each picture. (**a**) Italian strain *C. aegerita* CBS 358.51. (**b**) *C. aegerita* AaM isolated from *C. aegerita* mushrooms commercially acquired in a US supermarket. (**c**) Genome-sequenced strain *C. aegerita* AAE-3 derived from the reportedly Italian strain *C. aegerita* 4022. (**d**) Dutch strain *C. aegerita* CBS 127.88. (**e**) Italian strain *C. aegerita* DSM 9613. (**f**) Strain *C. aegerita* IHI536 isolated from *C. aegerita* mushrooms bought in an Italian supermarket. (**g**) German strain of *C. aegerita* (IHI8). (**h**) Strain *C. aegerita* CBS 832.87 of unknown origin. (**i**) English strain *C. aegerita* CBS 178.69. (**j**) Chinese strain *Cyclocybe* sp. MES02023. (**k**) Chinese strain *Cyclocybe* sp. IHI15. (**l**) Indian strain *Cyclocybe* sp. IHI392. (**m**) East German strain *Cyclocybe* sp. DSM 22459. (**n**) Thai strain of *Cyclocybe* sp. SC960903. (**o**) New Zealand strain *C. parasitica* ICMP 16333. (**p**) New Zealand strain *C. parasitica* ICMP 11668. (**q**) German strain C. *erebia* IHI606. Bar = 65 μm. (**r**) Strain *A. firma* CBS 390.79 (unknown origin) grown for 14 days. Bar = 11 μm. (**s**) German strain *A. arvalis* DSM 9710 grown for 14 days (PNG 3389 kb)

High Resolution (TIF 4356 kb)

Fig. S5 Fruiting patterns of different *Cyclocybe aegerita* strains (illustrated by three representative pictures per strain) in the fruiting setup of Herzog et al. (2016), 24–50 days post inoculation (pre-incubation, pi: 11–13 d at 25 °C in the dark; fruiting induction, fi: 11–39 d at 20 °C 12 h light/12 h dark). If not specified differently, red arrows point to stunted immature primordia. (**a**) Italian strain *C. aegerita* CBS 358.51, 13 d pi, 11 d fi; right photo: 15 d fi. B. Strain *C. aegerita* AaM isolated from *C. aegerita* mushrooms bought in a US supermarket, 12 d pi, 13 d fi; right photo: a red arrow points to a stunted immature basidiome (FB). (**c**) Genome-sequenced strain *C. aegerita* AAE-3 derived from the reportedly Italian strain *C. aegerita* 4022, 11 d pi, 14 d fi; central photo: 17 fi; right photo: 23 d fi, red arrows point to a stunted immature FB. (**d**) Dutch strain *C. aegerita* CBS 127.88, 11 d pi, 39 d fi. Bar = 2 cm (PNG 3931 kb)

High Resolution (TIF 4516 kb)

Fig. S6 Fruiting patterns of different *Cyclocybe aegerita* strains (illustrated by three representative pictures per strain) in the fruiting setup of Herzog et al. (2016), 25–47 days post inoculation (pre-incubation, pi: 11–13 d at 25 °C in the dark; fruiting induction, fi: 12–36 d at 20 °C 12 h light/12 h dark). If not specified differently, red arrows point to stunted immature basidiomes. (**a**) Italian strain *C. aegerita* DSM 9613, 12 d pi, 13 d fi. (b) Strain *C. aegerita* IHI536 isolated from *C. aegerita* mushrooms bought in an Italian supermarket, 11 d pi, 14 d fi. (**c**) German strain *C. aegerita* IHI8, 11 d pi, 36 d fi; left photo: 14 d fi; right photo: 27 d fi. (**d**) Strain *C. aegerita* CBS 832.87 the origin of which is unknown, 13 d pi, 12 d fi; right photo: a red arrow points to a stunted primordium. Bar = 2 cm (PNG 3710 kb)

High Resolution (TIF 4161 kb)

Fig. S7 Fruiting patterns of one strain of *Cyclocybe aegerita* and three strains from the Asian monophylum/monophyletic species complex preliminarily named *C. chaxingu* agg. (illustrated by three representative pictures per strain), if not specified differently, in the fruiting setup of Herzog et al. (2016), 28–55 days post inoculation (pre-incubation, pi: 11–14 d at 25 °C in the dark; fruiting induction, fi: 17–41 d at 20 °C 12 h light/12 h dark). (**a**) English strain *C. aegerita* CBS 178.69, 11 d pi, 17 d fi; right photo: a red arrow points to stunted primordia. (**b**) Chinese strain *Cyclocybe* sp. MES02023, 14 d pi at 22 °C, 32 d fi at 26 °C. (**c**) Indian strain *Cyclocybe* sp. IHI392, 13 d pi, 19 d fi. (**d**) Thai strain *Cyclocybe* sp. SC960903, 14 d pi at 30 °C, 41 d fi at 26 °C. Bar = 2 cm (PNG 4218 kb)

High Resolution (TIF 4844 kb)

Fig. S8 Fruiting patterns of *Cyclocybe parasitica* (illustrated by three representative pictures per strain) in the fruiting setup of Herzog et al. (2016), 34–50 days post inoculation (pre-incubation, pi: 13–15 d at 25 °C in the dark; fruiting induction, fi: 21–35 d at 20 °C 12 h light/12 h dark). (**a**) New Zealand strain *C. parasitica* ICMP 16333, 15 d pi, 35 d fi; right photo: 32 d fi. (**b**) New Zealand strain *C. parasitica* ICMP 11668, 13 d pi, 21 d fi; central photo: 23 d fi; left photo: 25 d fi. Bar = 2 cm. (PNG 2176 kb)

High Resolution (TIF 2573 kb)

Fig. S9 Fruiting features of one *C. aegerita* strain and two strains from the Asian monophylum/monophyletic species complex preliminarily named *C. chaxingu* agg. in the fruiting setup of Herzog et al. (2016) normally comprising (**a**–**c)** a vegetative growth (pre-incubation) temperature of 25 °C, and a fruiting temperature of 20 °C. This was changed to (**d**–**f**) 30 °C and 26 °C or (**g**–**h**) to 22 °C and 26 °C. (**a**) Genome-sequenced strain *C. aegerita* AAE-3 derived from the reportedly Italian strain *C. aegerita* 4022, after 11 days pre-incubation, pi and 14 days fruiting induction, fi. (**b**) Northern Chinese strain *Cyclocybe* sp. MES02023 from the Jilin Province, 12 d pi, 33 d fi. (**c**) Thai strain *Cyclocybe* sp. SC960903, 11 d pi, 35 d fi. (**d**) *C. aegerita* AAE-3, 14 d pi, 35 d fi. (**e**) *Cyclocybe* sp. MES02023, 21 d pi, 35 d fi. (**f**) *Cyclocybe* sp. SC960903, 14 d pi, 39 d fi. (**g**) *Cyclocybe* sp. MES02023, 14 d pi, 33 d fi. (**h**) *Cyclocybe* sp. SC960903, 10 d pi, 35 d fi. Bar = 2 cm (PNG 3149 kb)

High Resolution (TIF 3629 kb)

Supplementary Table S1 *TEF1α* and *RPB2* primers employed in this study. (XLS) (XLS 21 kb)

Supplementary Table S2 Details of all reference strains used in this study. (XLS) (XLS 53 kb)

Supplementary Table S3 Fruiting productivity of “*Cyclocybe aegerita* sensu *lato*” and *C. parasitica* in the fruiting setup of Herzog et al. (2016). (XLS) (XLS 24 kb)

Online Resource 1 Concatenated alignment of ITS and LSU sequences of *Agrocybe* and *Cyclocybe* species as well as of selected species of Cortinariaceae, Hymenogastraceae, Mycenaceae, Schizophyllaceae, and Strophariaceae. (NEX) (NEX 107 kb)

Online Resource 2 Concatenated alignment of ITS, LSU, *RPB2* and *TEF1α* sequences of *Agrocybe* and *Cyclocybe* species for which these sequences were available or generated in the present study. (NEX) (NEX 70 kb)
