# Supplementary figures and images for: Multilocus phylogeny- and fruiting feature-assisted delimitation of European Cyclocybe aegerita from a new Asian species complex and related species
Source: Mycol Prog. 2020 Oct 8;19(10):1001–16. doi: 10.1007/s11557-020-01599-z (PMC7541202; doi:10.1007/s11557-020-01599-z)

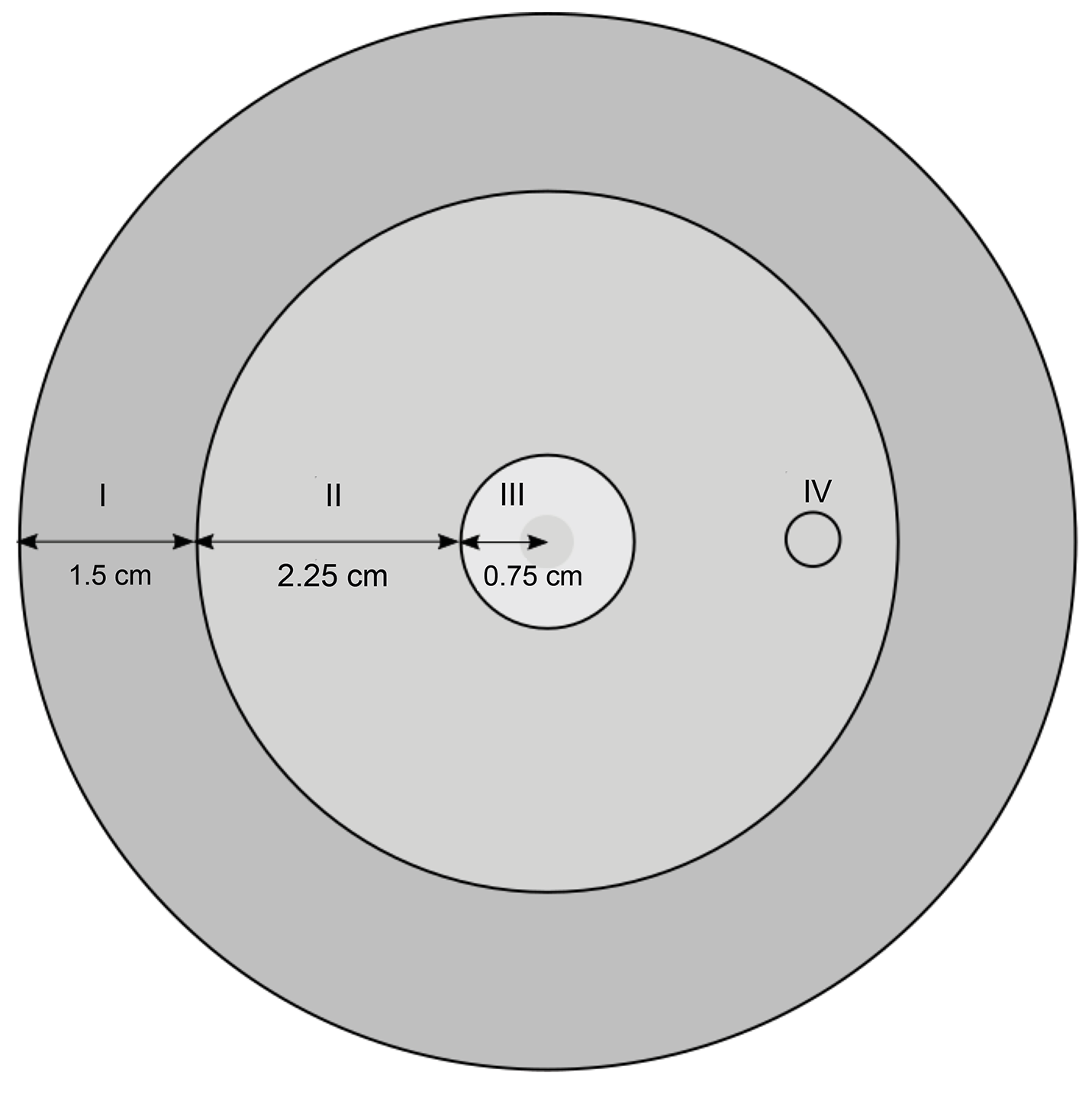

Supplement: Supplementary file 1 — Subdivision of the cultivation mediums surface into different zones. The edge zone (I) spans 1.5 cm starting from the plate edge and ending at the distal end of the periphery zone (II). The periphery zone measures 2.25 cm from the end of the edge zone edge towards the plate centre, ending at the distal end of the centre zone. The centre zone (III) covers 0.75 cm from the plate centre towards the periphery zone. The point-of-injury zone (IV) is made up by a 0.5 cm2-punched-out hole in the distal area of the periphery zone (PNG 244 kb) [file 11557_2020_1599_Fig6_ESM.png]

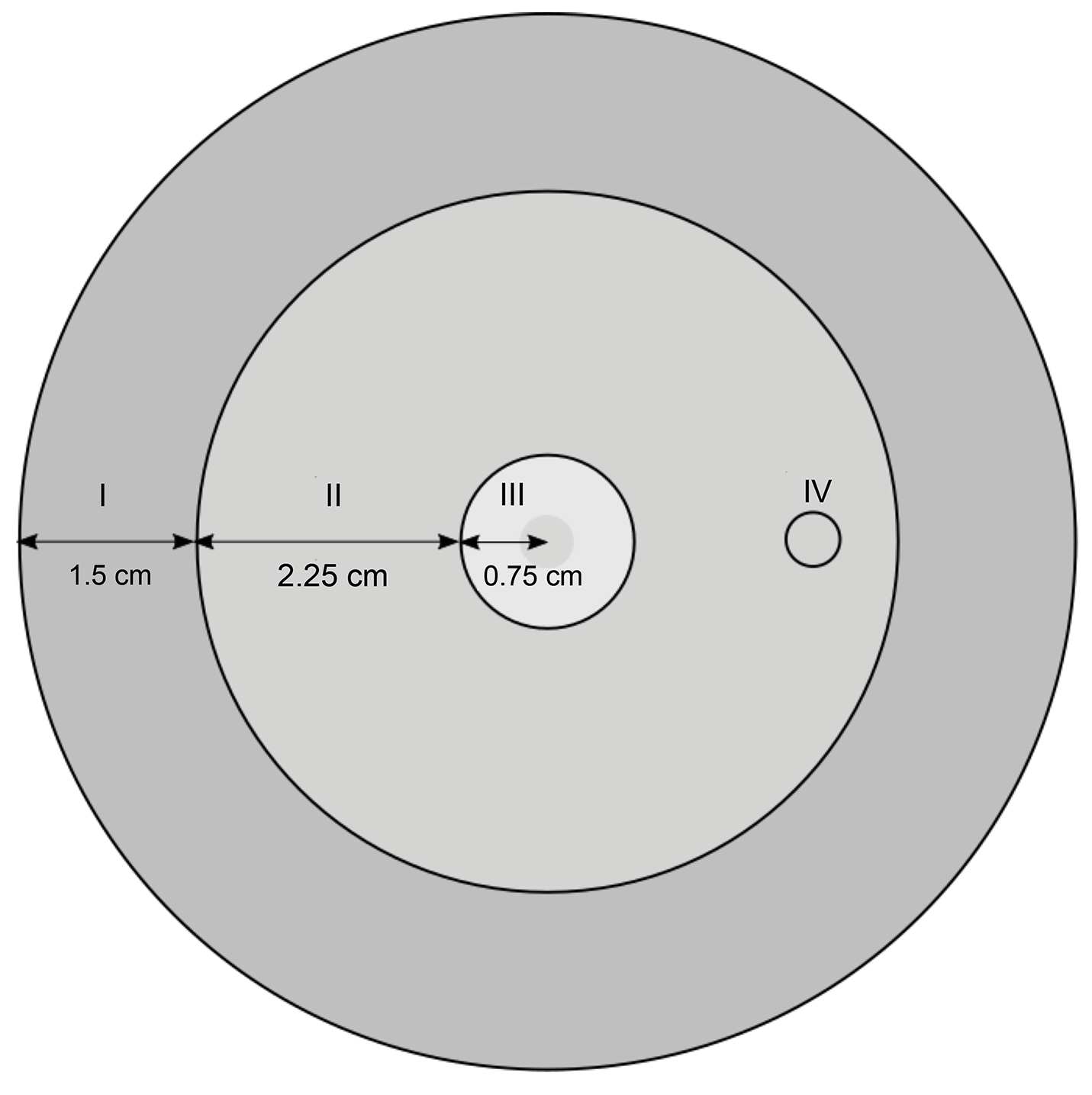

Supplement: Supplementary file 2 — High Resolution (TIF 282 kb) [file 11557_2020_1599_MOESM1_ESM.tif]

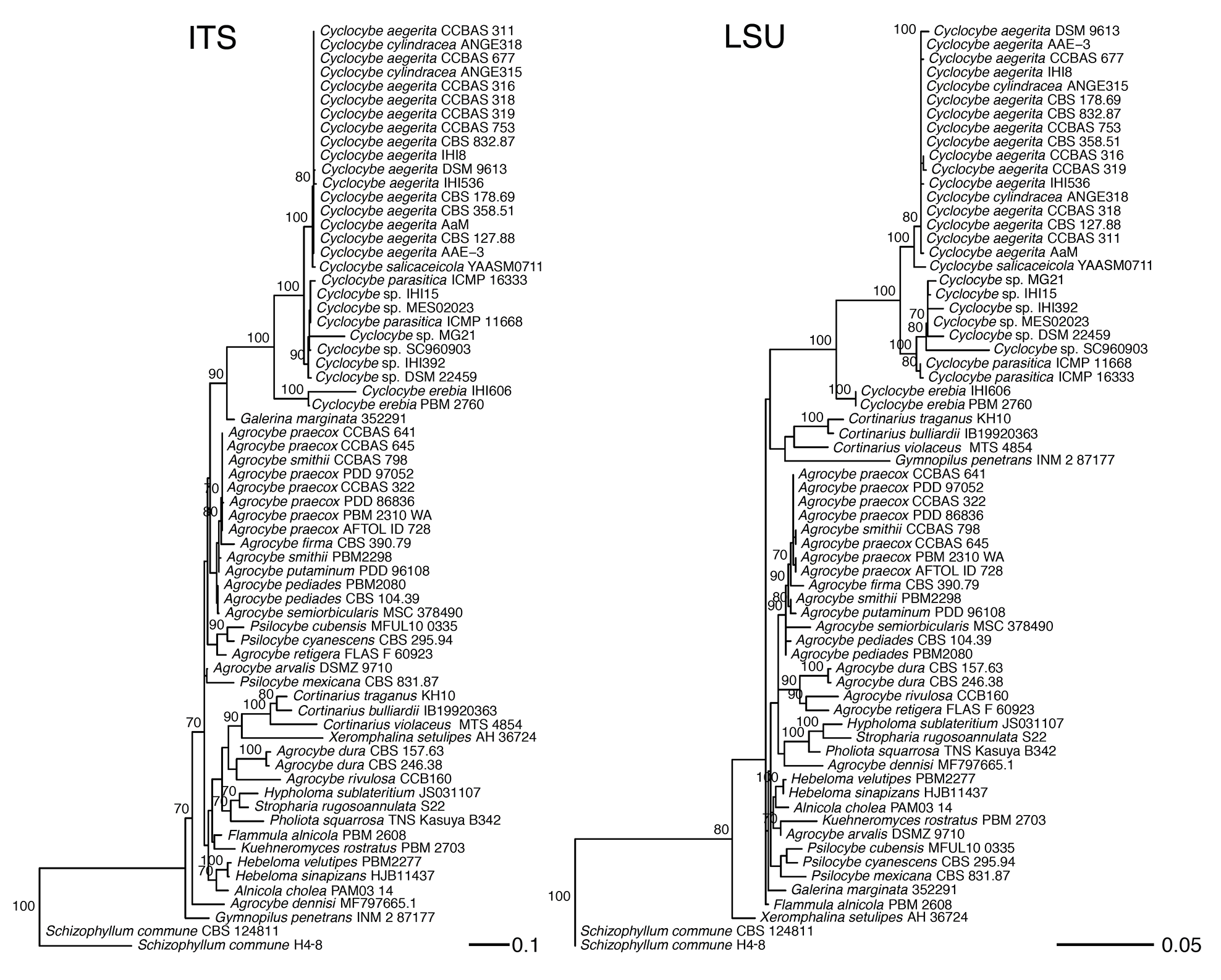

Supplement: Supplementary file 3 — Maximum likelihood (ML) trees of Agrocybe spp. and Cyclocybe spp. towards a selection of hymenogastraceous or strophariaceous Agaricales taxa, based on a ITS or LSU sequences. Support values above the branches: ML bootstrap value (BT) in absolute numbers. Only support values of BT ≥ 70 are displayed for each node (PNG 1018 kb) [file 11557_2020_1599_Fig7_ESM.png]

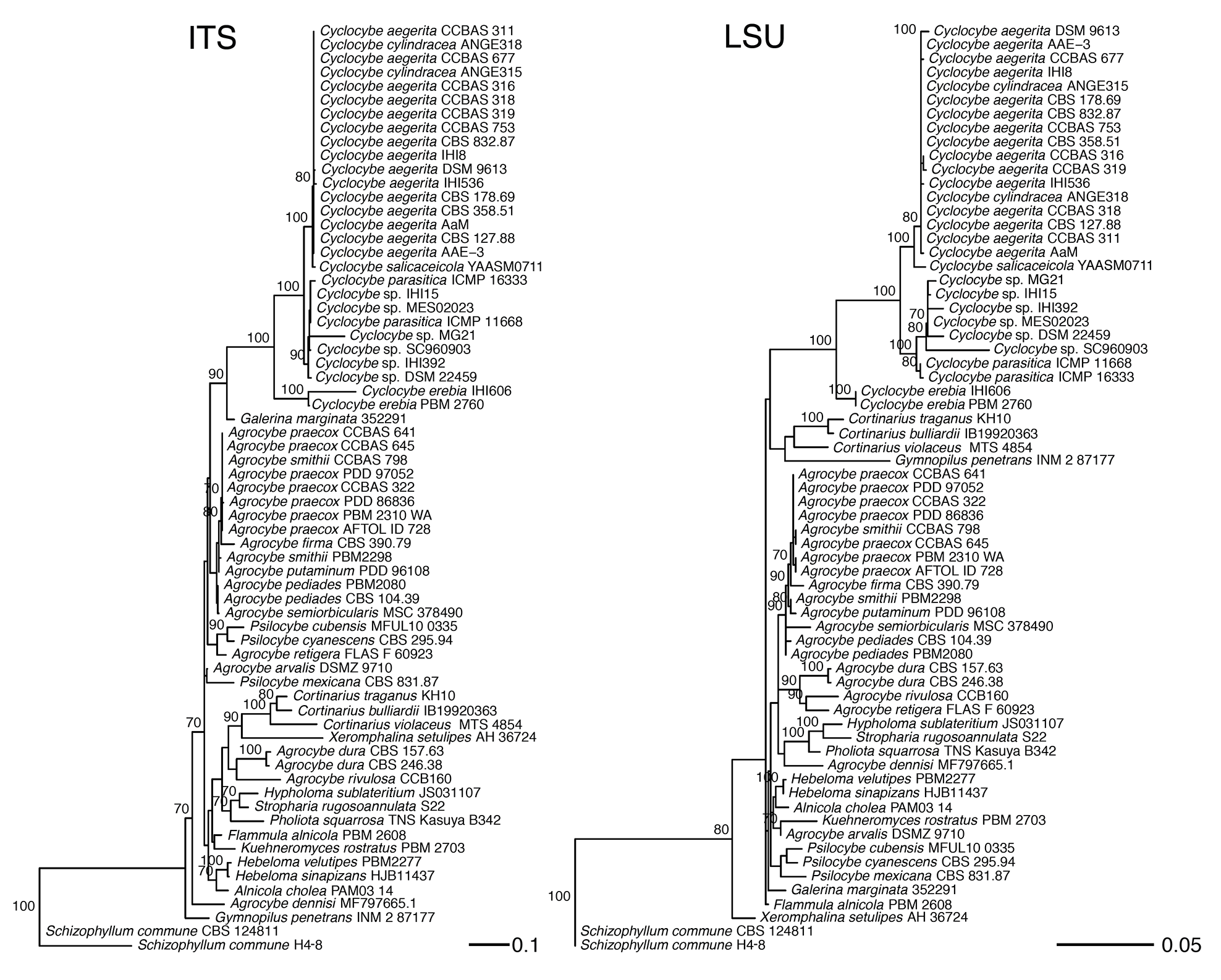

Supplement: Supplementary file 4 — High Resolution (TIF 1232 kb) [file 11557_2020_1599_MOESM2_ESM.tif]

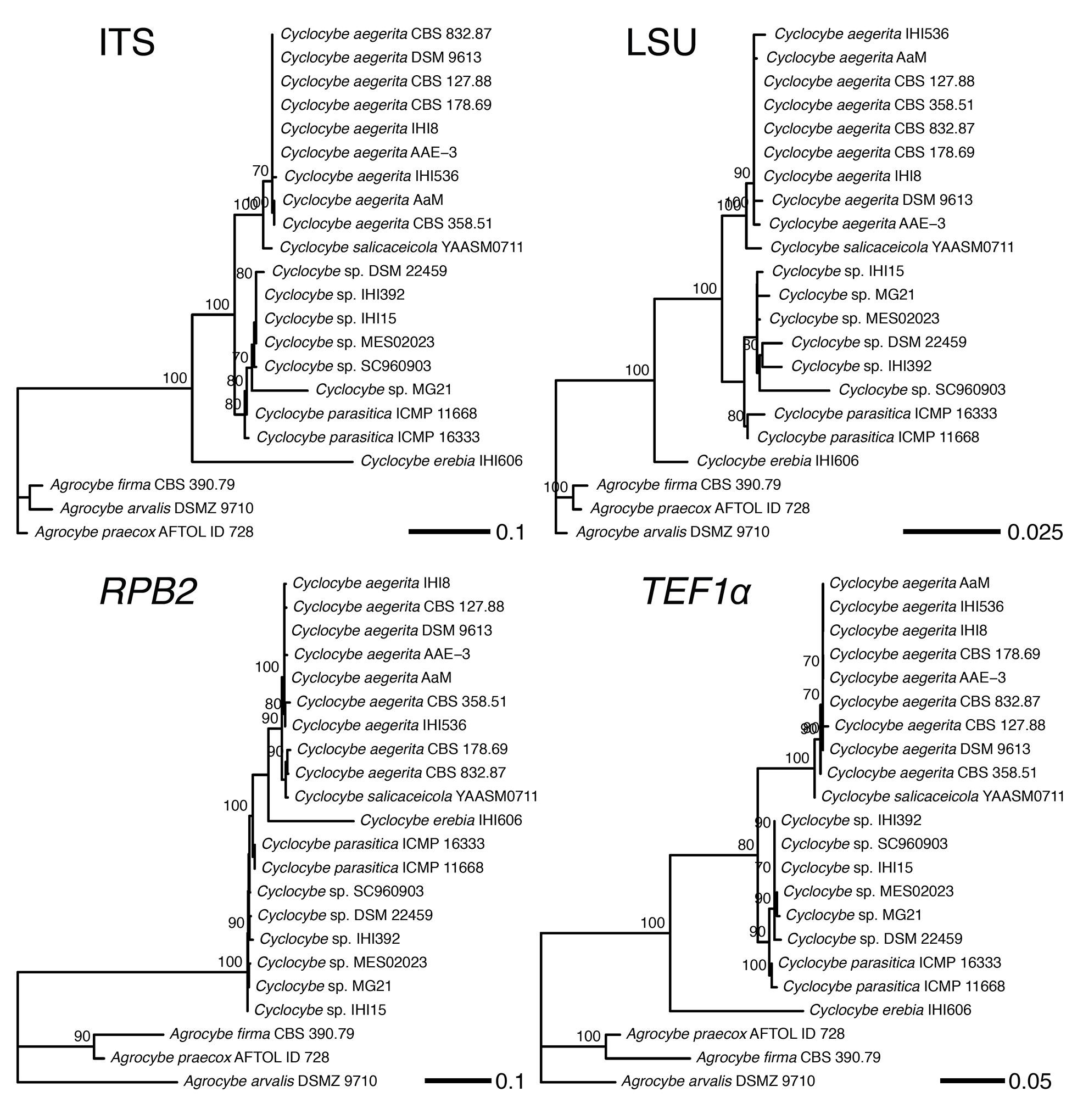

Supplement: Supplementary file 5 — Maximum likelihood (ML) trees of Agrocybe spp. and Cyclocybe spp., based on ITS, LSU, RPB2, or TEF1α sequences. Support values above the branches: ML bootstrap value (BT) in absolute numbers. Only support values of BT ≥ 70 are displayed for each node (PNG 629 kb) [file 11557_2020_1599_Fig8_ESM.png]

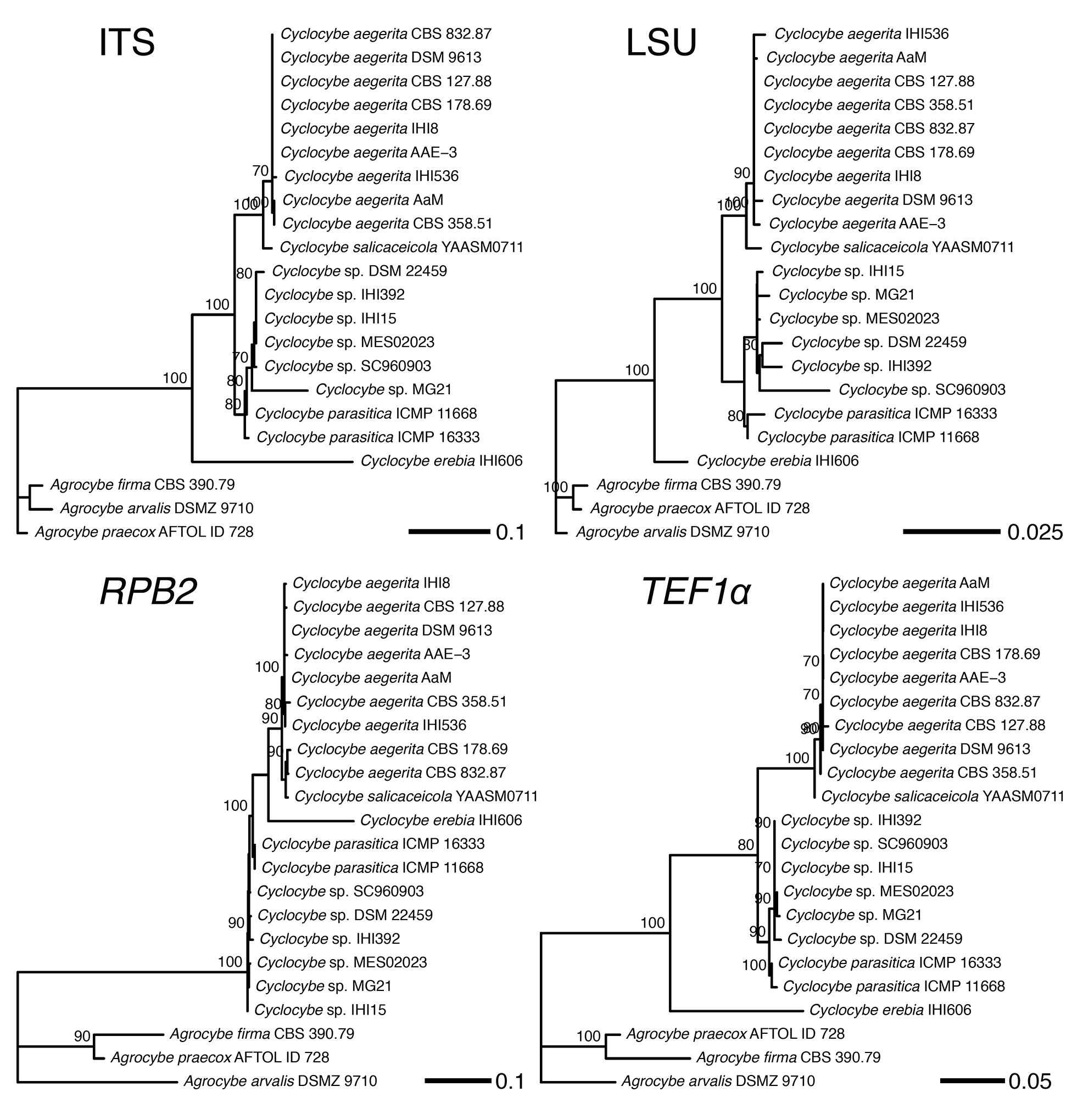

Supplement: Supplementary file 6 — High Resolution (TIF 966 kb) [file 11557_2020_1599_MOESM3_ESM.tif]

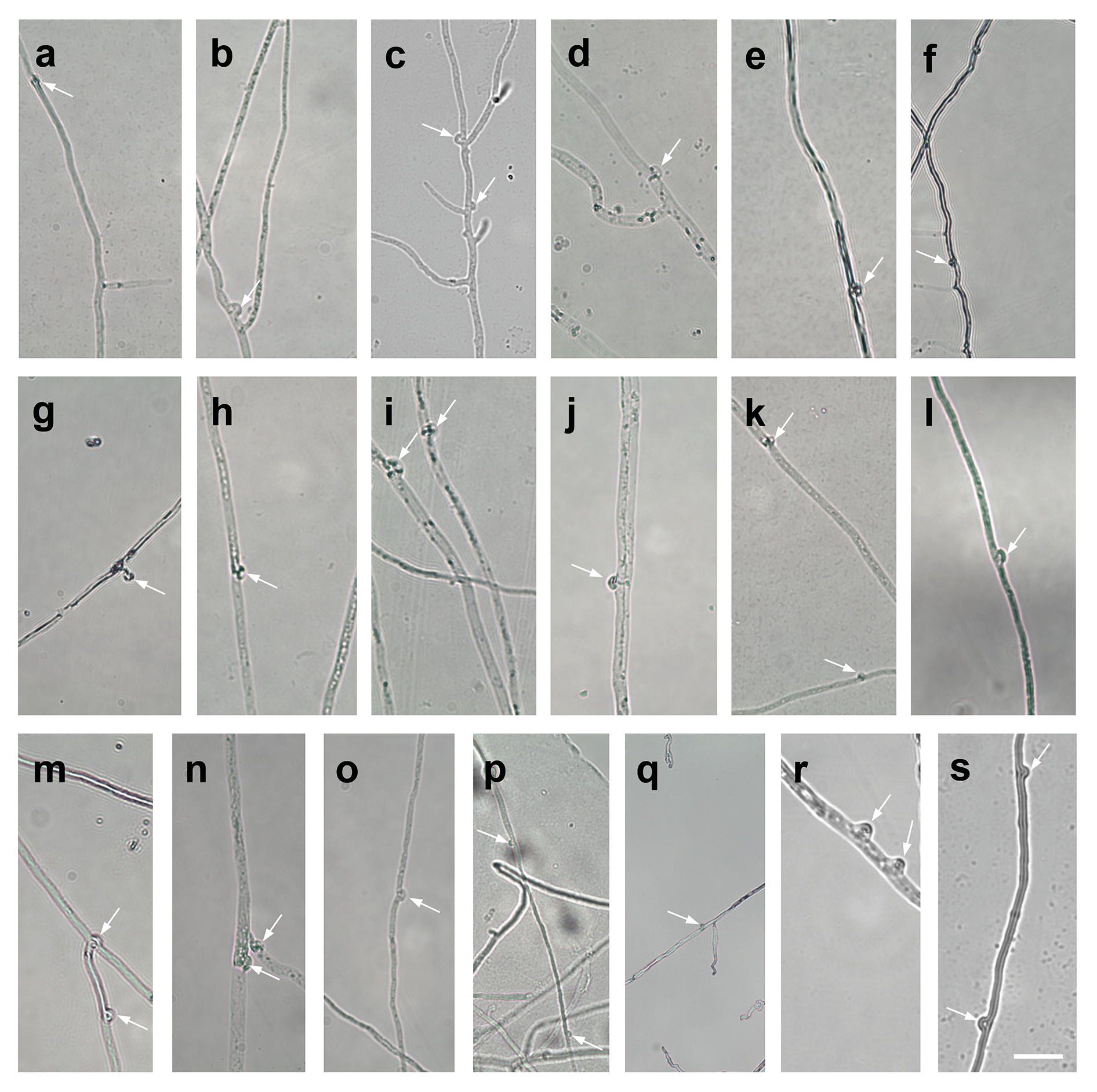

Supplement: Supplementary file 7 — Clamp formation by different dikaryotic strains of Cyclocybe aegerita, from the Asian monophylum/monophyletic species complex preliminarily named C. chaxingu agg., and from C. parasitica, if not specified differently, grown in a micro-cultivation chamber for 7 days at 25 °C. If not specified differently, bar = 20 μm. White arrows mark the position of clamp connections in each picture. (a) Italian strain C. aegerita CBS 358.51. (b) C. aegerita AaM isolated from C. aegerita mushrooms commercially acquired in a US supermarket. (c) Genome-sequenced strain C. aegerita AAE-3 derived from the reportedly Italian strain C. aegerita 4022. (d) Dutch strain C. aegerita CBS 127.88. (e) Italian strain C. aegerita DSM 9613. (f) Strain C. aegerita IHI536 isolated from C. aegerita mushrooms bought in an Italian supermarket. (g) German strain of C. aegerita (IHI8). (h) Strain C. aegerita CBS 832.87 of unknown origin. (i) English strain C. aegerita CBS 178.69. (j) Chinese strain Cyclocybe sp. MES02023. (k) Chinese strain Cyclocybe sp. IHI15. (l) Indian strain Cyclocybe sp. IHI392. (m) East German strain Cyclocybe sp. DSM 22459. (n) Thai strain of Cyclocybe sp. SC960903. (o) New Zealand strain C. parasitica ICMP 16333. (p) New Zealand strain C. parasitica ICMP 11668. (q) German strain C. erebia IHI606. Bar = 65 μm. (r) Strain A. firma CBS 390.79 (unknown origin) grown for 14 days. Bar = 11 μm. (s) German strain A. arvalis DSM 9710 grown for 14 days (PNG 3389 kb) [file 11557_2020_1599_Fig9_ESM.png]

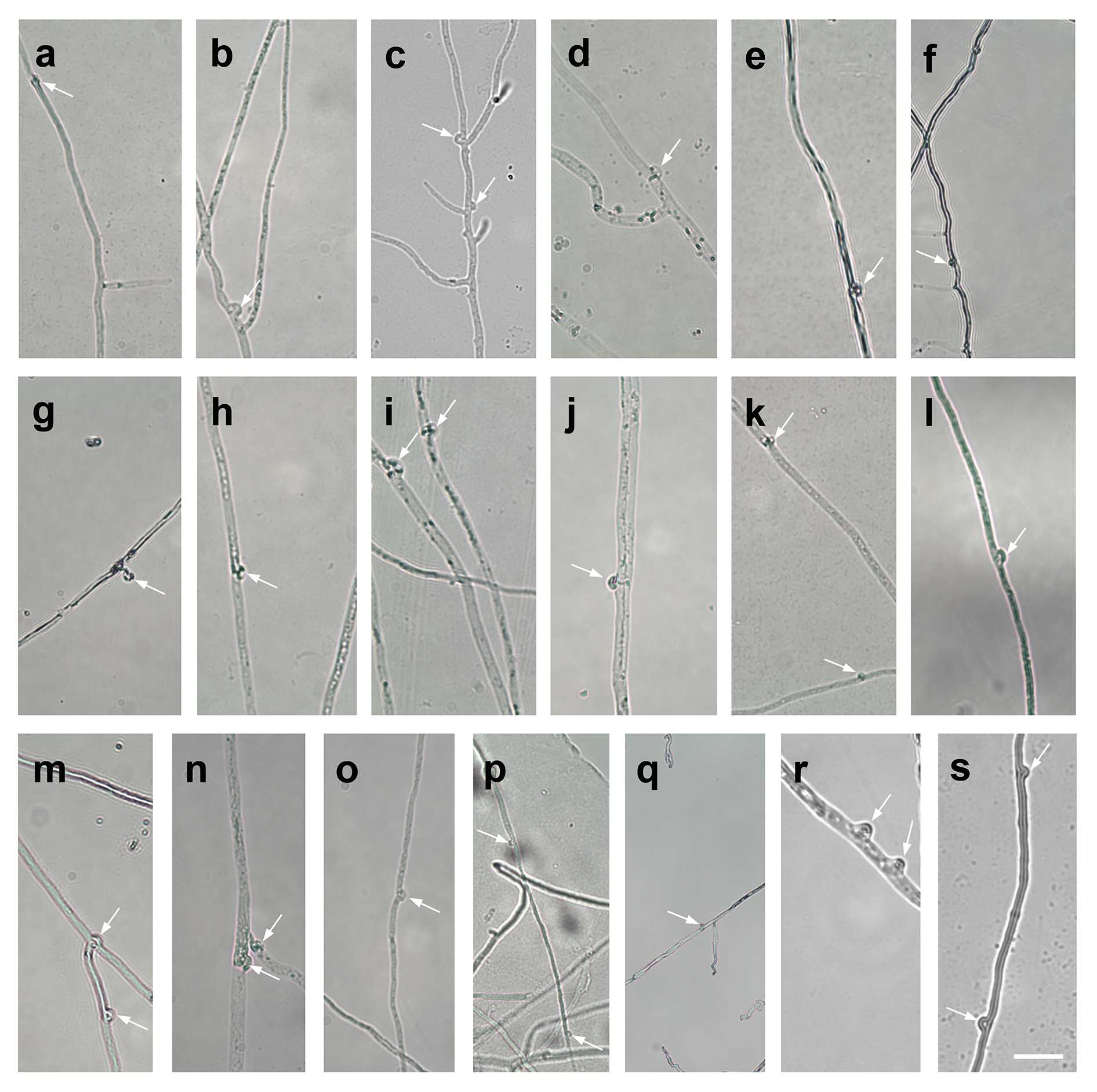

Supplement: Supplementary file 8 — High Resolution (TIF 4356 kb) [file 11557_2020_1599_MOESM4_ESM.tif]

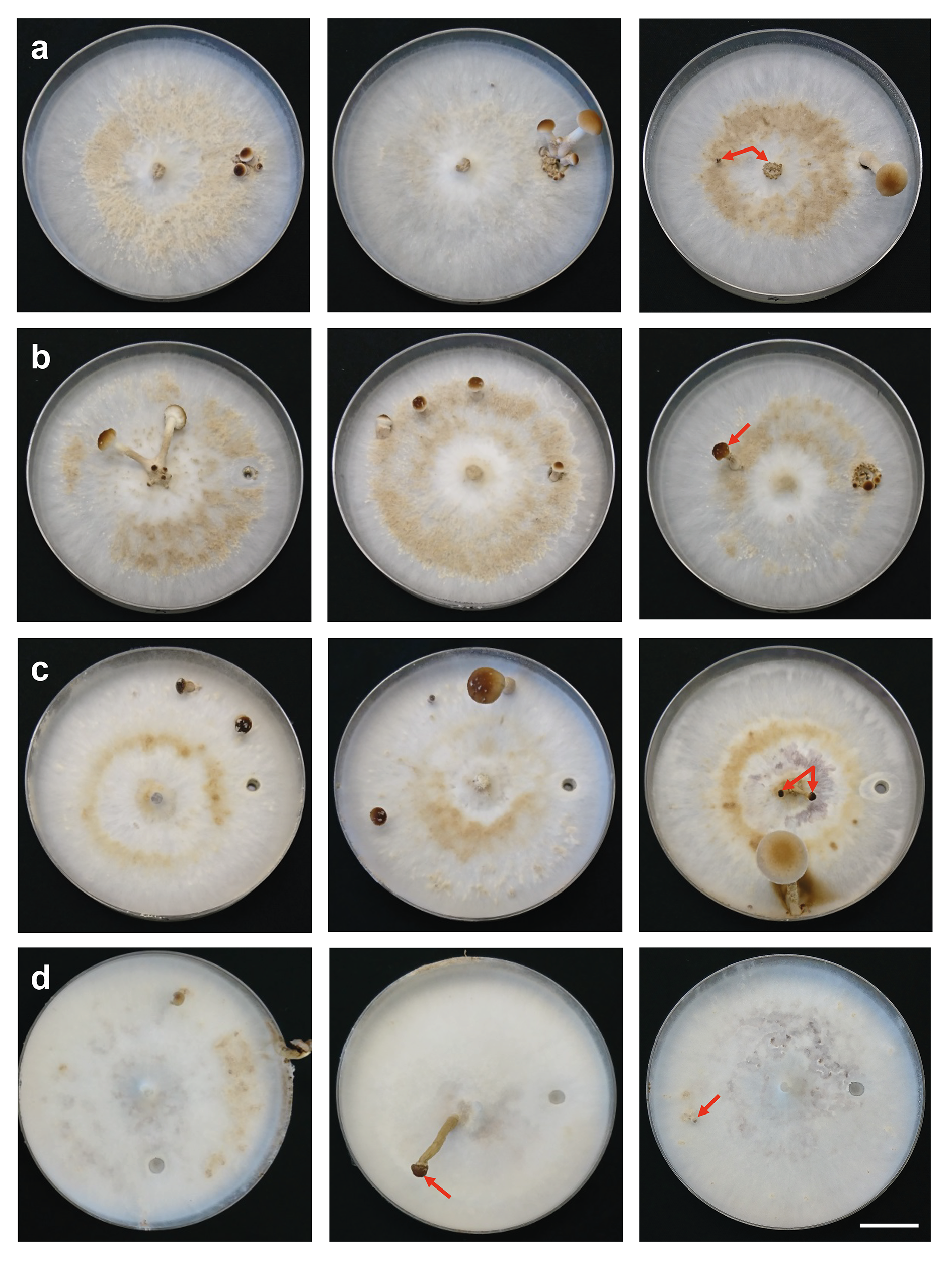

Supplement: Supplementary file 9 — Fruiting patterns of different Cyclocybe aegerita strains (illustrated by three representative pictures per strain) in the fruiting setup of Herzog et al. (2016), 24–50 days post inoculation (pre-incubation, pi: 11–13 d at 25 °C in the dark; fruiting induction, fi: 11–39 d at 20 °C 12 h light/12 h dark). If not specified differently, red arrows point to stunted immature primordia. (a) Italian strain C. aegerita CBS 358.51, 13 d pi, 11 d fi; right photo: 15 d fi. B. Strain C. aegerita AaM isolated from C. aegerita mushrooms bought in a US supermarket, 12 d pi, 13 d fi; right photo: a red arrow points to a stunted immature basidiome (FB). (c) Genome-sequenced strain C. aegerita AAE-3 derived from the reportedly Italian strain C. aegerita 4022, 11 d pi, 14 d fi; central photo: 17 fi; right photo: 23 d fi, red arrows point to a stunted immature FB. (d) Dutch strain C. aegerita CBS 127.88, 11 d pi, 39 d fi. Bar = 2 cm (PNG 3931 kb) [file 11557_2020_1599_Fig10_ESM.png]

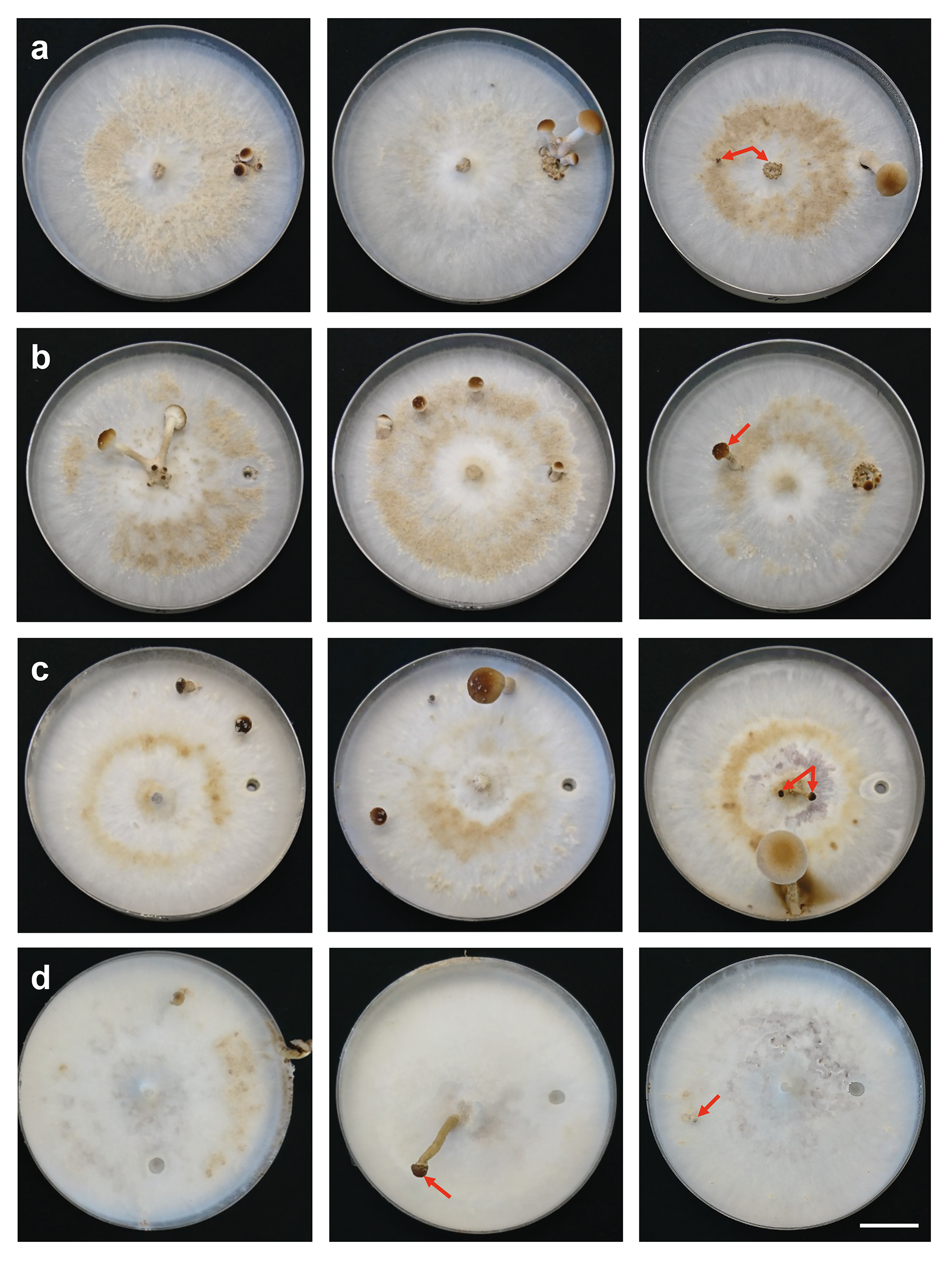

Supplement: Supplementary file 10 — High Resolution (TIF 4516 kb) [file 11557_2020_1599_MOESM5_ESM.tif]

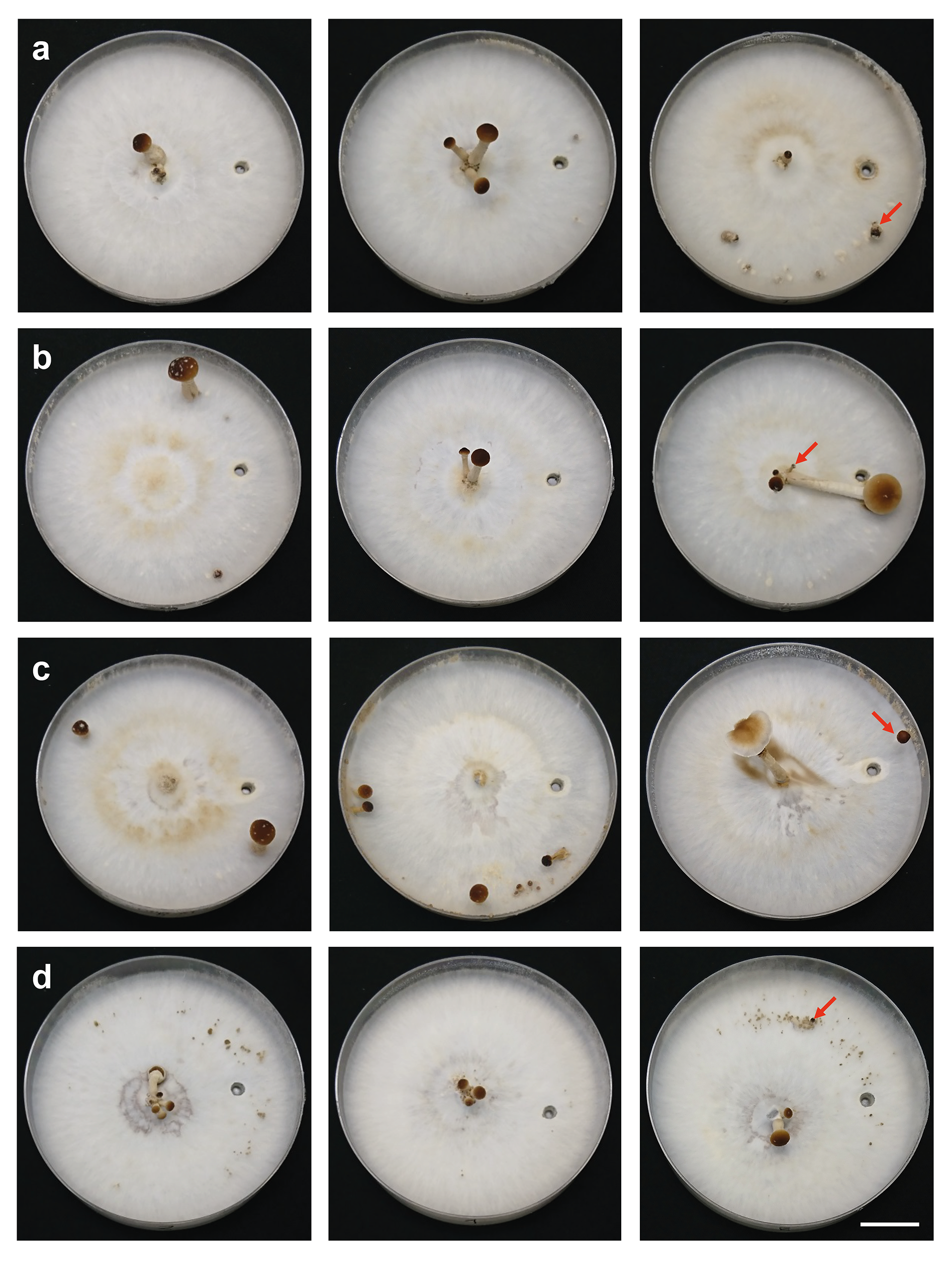

Supplement: Supplementary file 11 — Fruiting patterns of different Cyclocybe aegerita strains (illustrated by three representative pictures per strain) in the fruiting setup of Herzog et al. (2016), 25–47 days post inoculation (pre-incubation, pi: 11–13 d at 25 °C in the dark; fruiting induction, fi: 12–36 d at 20 °C 12 h light/12 h dark). If not specified differently, red arrows point to stunted immature basidiomes. (a) Italian strain C. aegerita DSM 9613, 12 d pi, 13 d fi. (b) Strain C. aegerita IHI536 isolated from C. aegerita mushrooms bought in an Italian supermarket, 11 d pi, 14 d fi. (c) German strain C. aegerita IHI8, 11 d pi, 36 d fi; left photo: 14 d fi; right photo: 27 d fi. (d) Strain C. aegerita CBS 832.87 the origin of which is unknown, 13 d pi, 12 d fi; right photo: a red arrow points to a stunted primordium. Bar = 2 cm (PNG 3710 kb) [file 11557_2020_1599_Fig11_ESM.png]

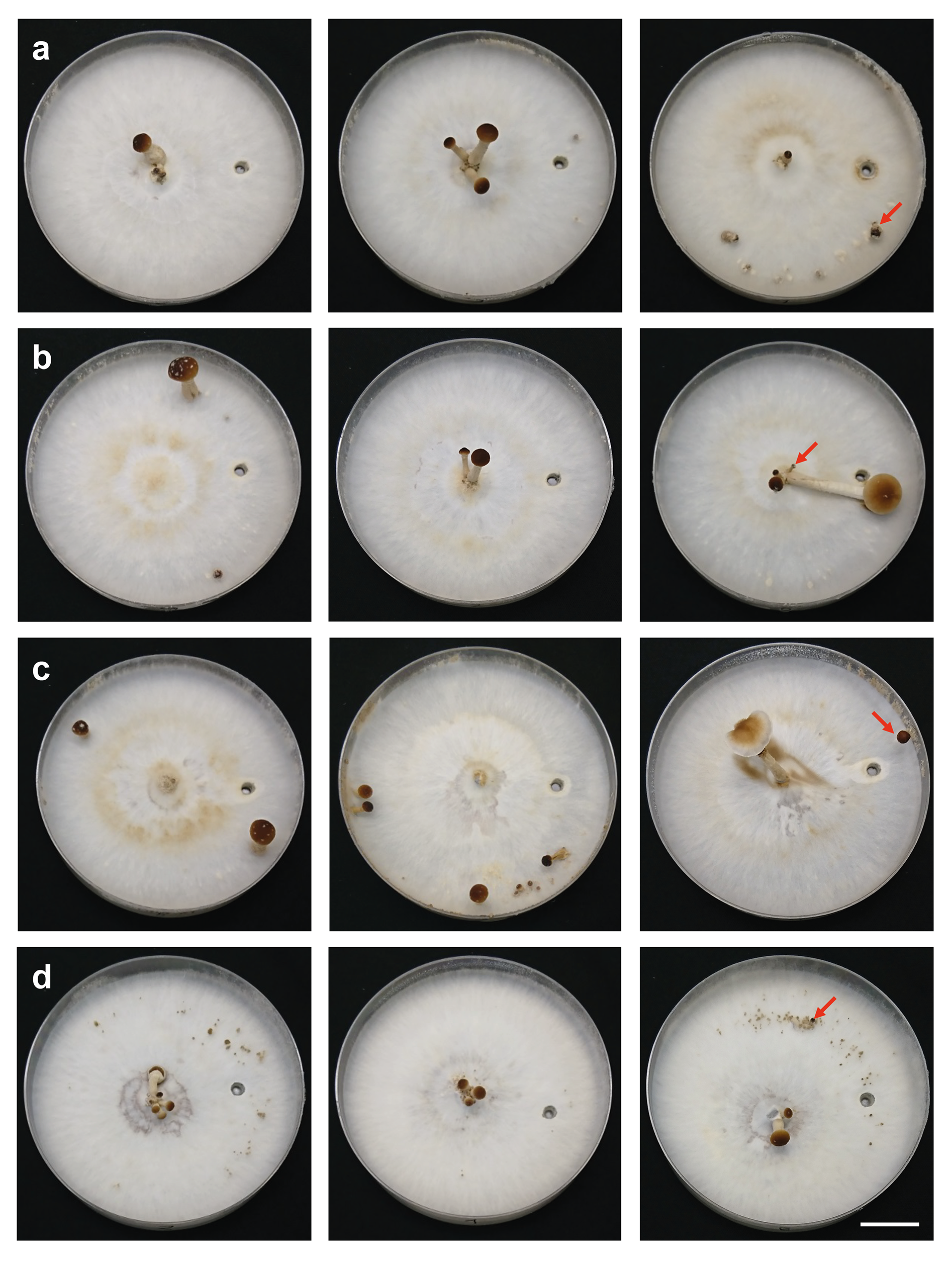

Supplement: Supplementary file 12 — High Resolution (TIF 4161 kb) [file 11557_2020_1599_MOESM6_ESM.tif]

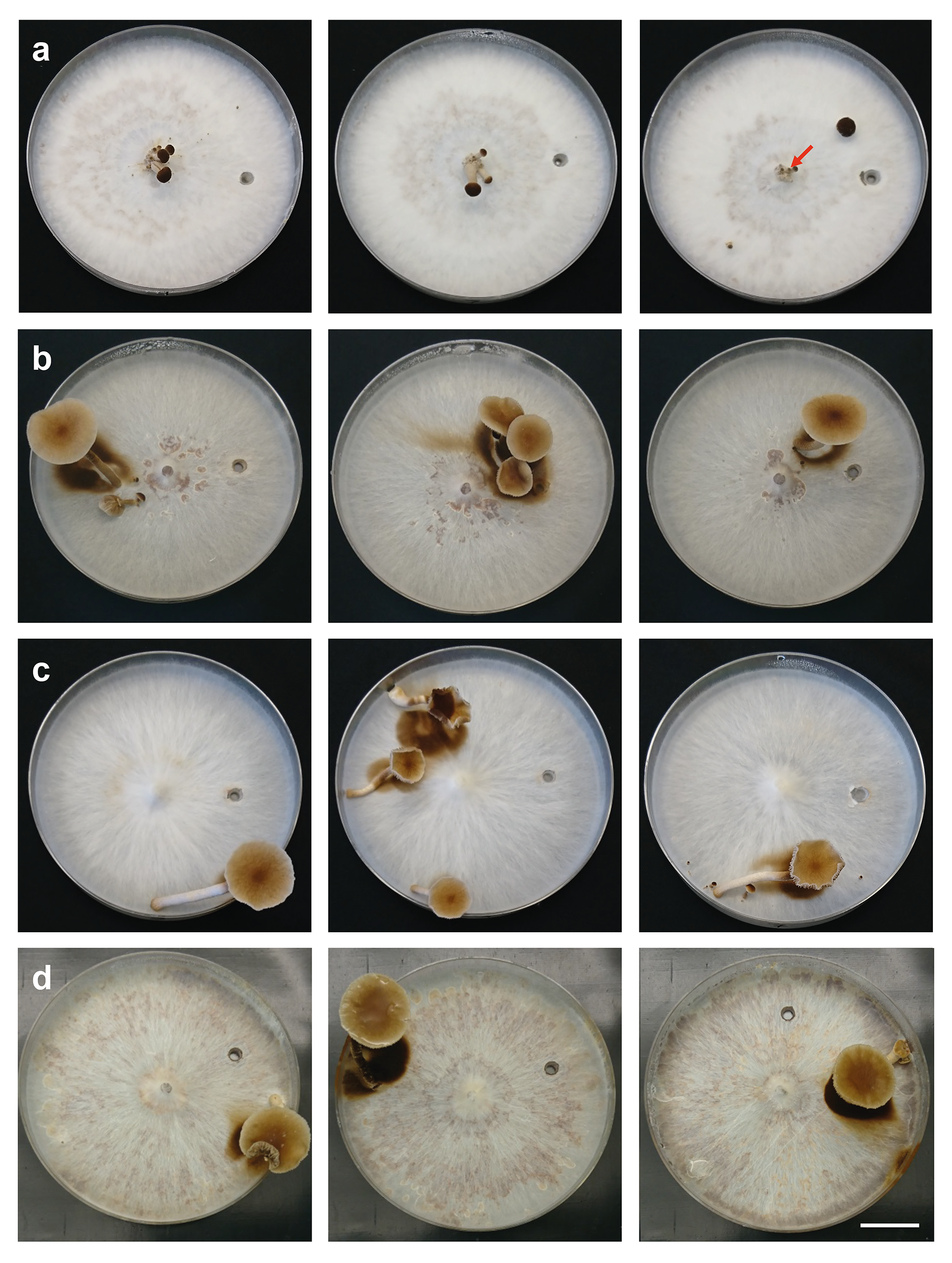

Supplement: Supplementary file 13 — Fruiting patterns of one strain of Cyclocybe aegerita and three strains from the Asian monophylum/monophyletic species complex preliminarily named C. chaxingu agg. (illustrated by three representative pictures per strain), if not specified differently, in the fruiting setup of Herzog et al. (2016), 28–55 days post inoculation (pre-incubation, pi: 11–14 d at 25 °C in the dark; fruiting induction, fi: 17–41 d at 20 °C 12 h light/12 h dark). (a) English strain C. aegerita CBS 178.69, 11 d pi, 17 d fi; right photo: a red arrow points to stunted primordia. (b) Chinese strain Cyclocybe sp. MES02023, 14 d pi at 22 °C, 32 d fi at 26 °C. (c) Indian strain Cyclocybe sp. IHI392, 13 d pi, 19 d fi. (d) Thai strain Cyclocybe sp. SC960903, 14 d pi at 30 °C, 41 d fi at 26 °C. Bar = 2 cm (PNG 4218 kb) [file 11557_2020_1599_Fig12_ESM.png]

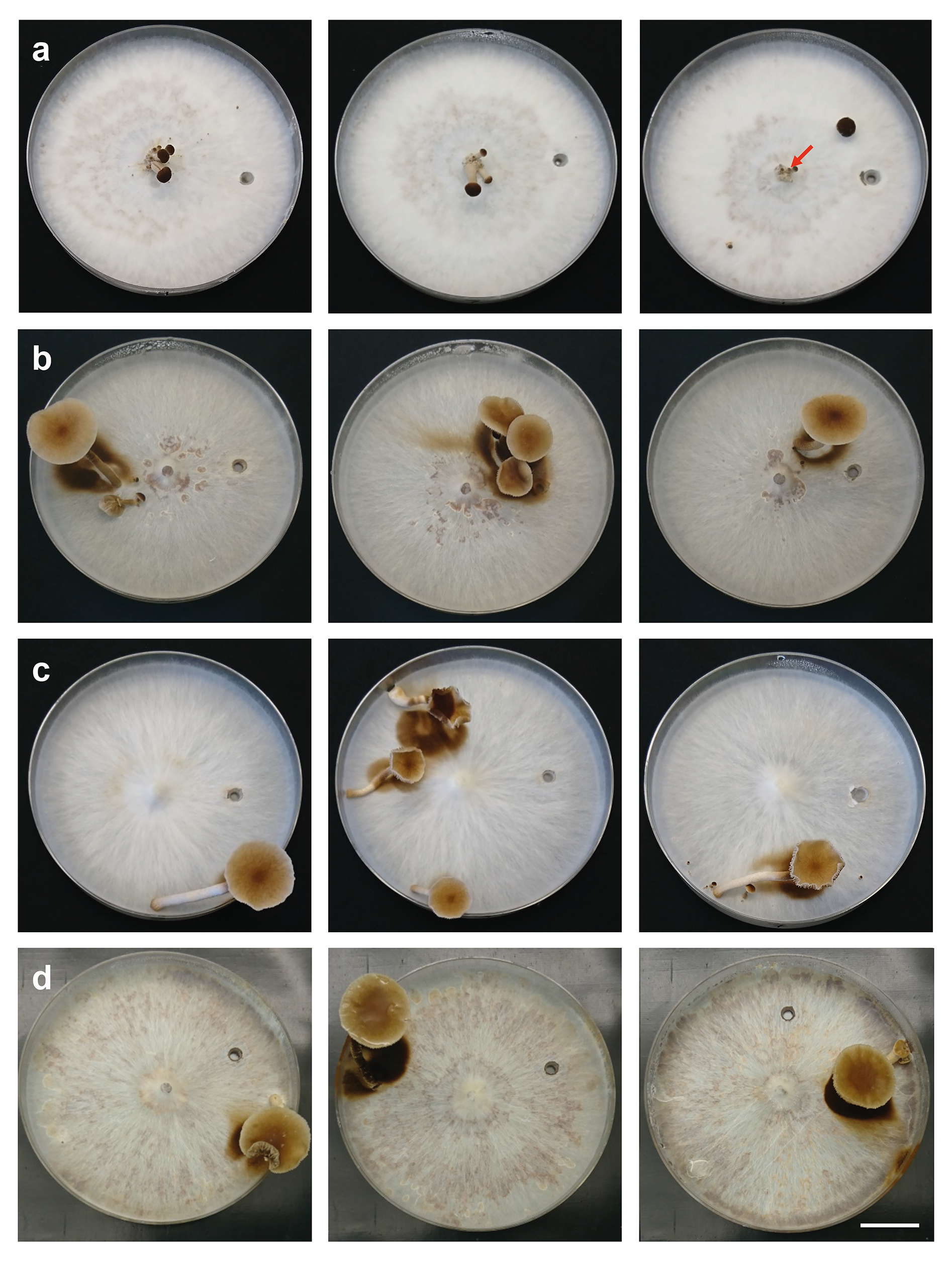

Supplement: Supplementary file 14 — High Resolution (TIF 4844 kb) [file 11557_2020_1599_MOESM7_ESM.tif]

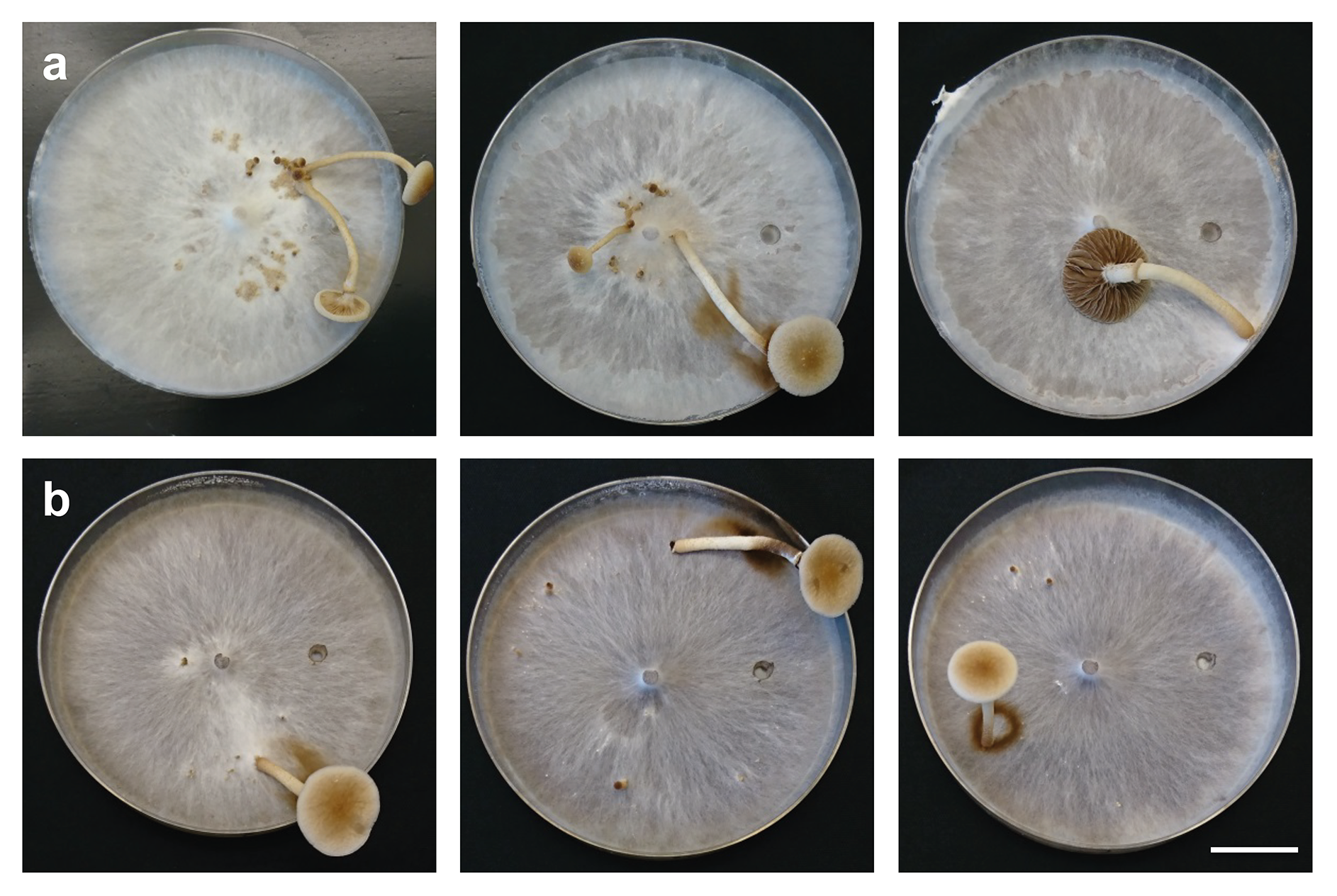

Supplement: Supplementary file 15 — Fruiting patterns of Cyclocybe parasitica (illustrated by three representative pictures per strain) in the fruiting setup of Herzog et al. (2016), 34–50 days post inoculation (pre-incubation, pi: 13–15 d at 25 °C in the dark; fruiting induction, fi: 21–35 d at 20 °C 12 h light/12 h dark). (a) New Zealand strain C. parasitica ICMP 16333, 15 d pi, 35 d fi; right photo: 32 d fi. (b) New Zealand strain C. parasitica ICMP 11668, 13 d pi, 21 d fi; central photo: 23 d fi; left photo: 25 d fi. Bar = 2 cm. (PNG 2176 kb) [file 11557_2020_1599_Fig13_ESM.png]

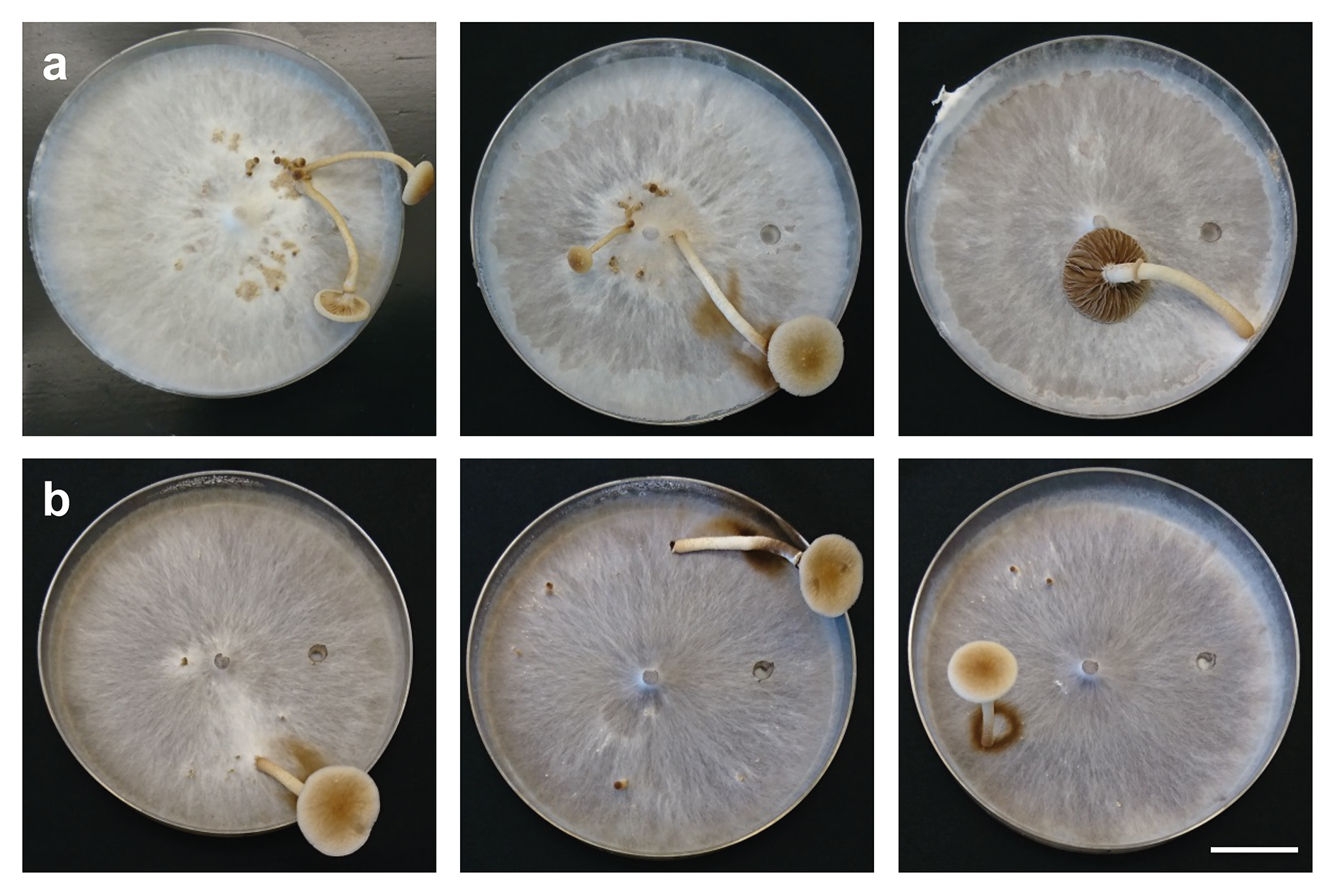

Supplement: Supplementary file 16 — High Resolution (TIF 2573 kb) [file 11557_2020_1599_MOESM8_ESM.tif]

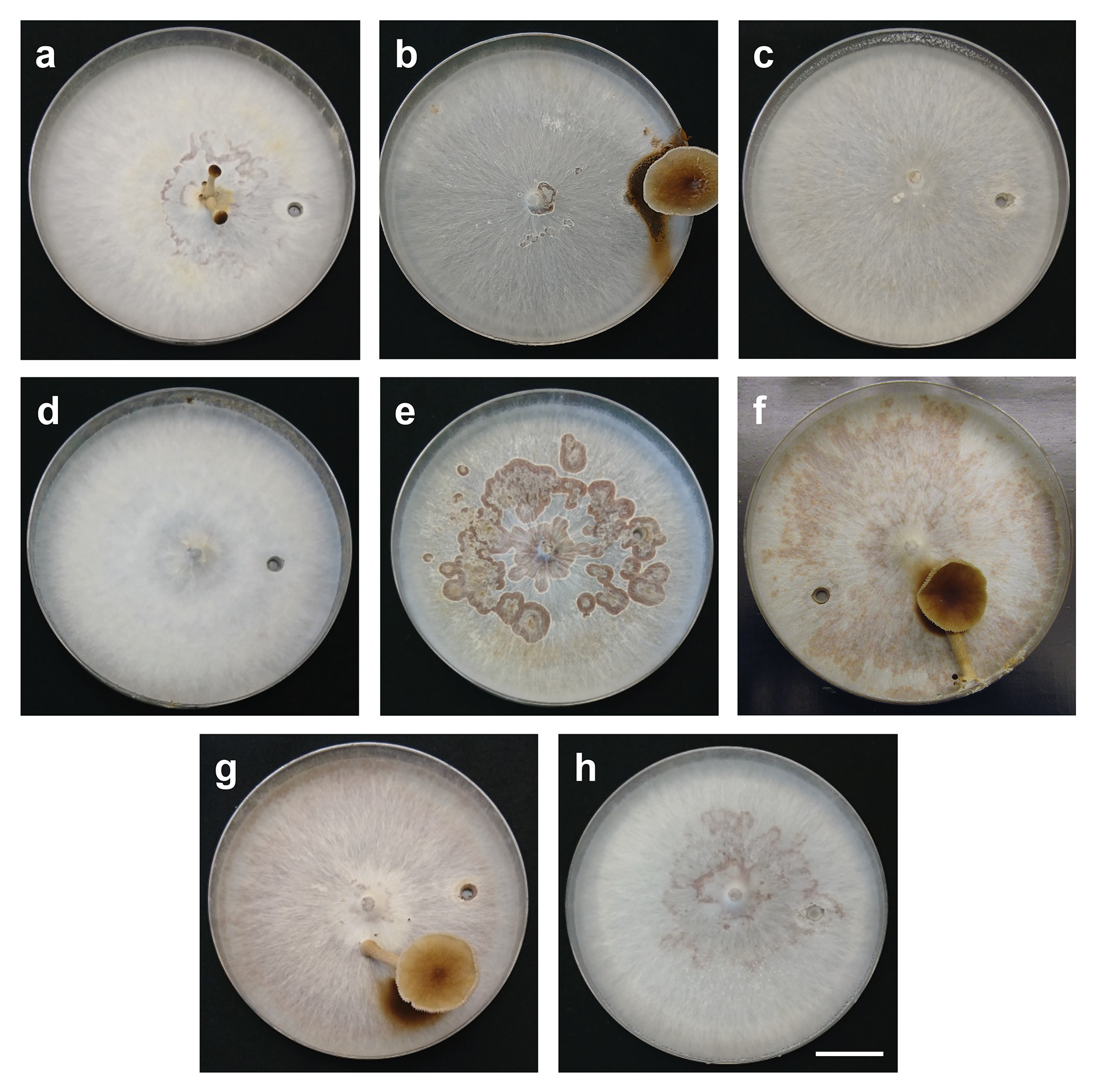

Supplement: Supplementary file 17 — Fruiting features of one C. aegerita strain and two strains from the Asian monophylum/monophyletic species complex preliminarily named C. chaxingu agg. in the fruiting setup of Herzog et al. (2016) normally comprising (a–c) a vegetative growth (pre-incubation) temperature of 25 °C, and a fruiting temperature of 20 °C. This was changed to (d–f) 30 °C and 26 °C or (g–h) to 22 °C and 26 °C. (a) Genome-sequenced strain C. aegerita AAE-3 derived from the reportedly Italian strain C. aegerita 4022, after 11 days pre-incubation, pi and 14 days fruiting induction, fi. (b) Northern Chinese strain Cyclocybe sp. MES02023 from the Jilin Province, 12 d pi, 33 d fi. (c) Thai strain Cyclocybe sp. SC960903, 11 d pi, 35 d fi. (d) C. aegerita AAE-3, 14 d pi, 35 d fi. (e) Cyclocybe sp. MES02023, 21 d pi, 35 d fi. (f) Cyclocybe sp. SC960903, 14 d pi, 39 d fi. (g) Cyclocybe sp. MES02023, 14 d pi, 33 d fi. (h) Cyclocybe sp. SC960903, 10 d pi, 35 d fi. Bar = 2 cm (PNG 3149 kb) [file 11557_2020_1599_Fig14_ESM.png]

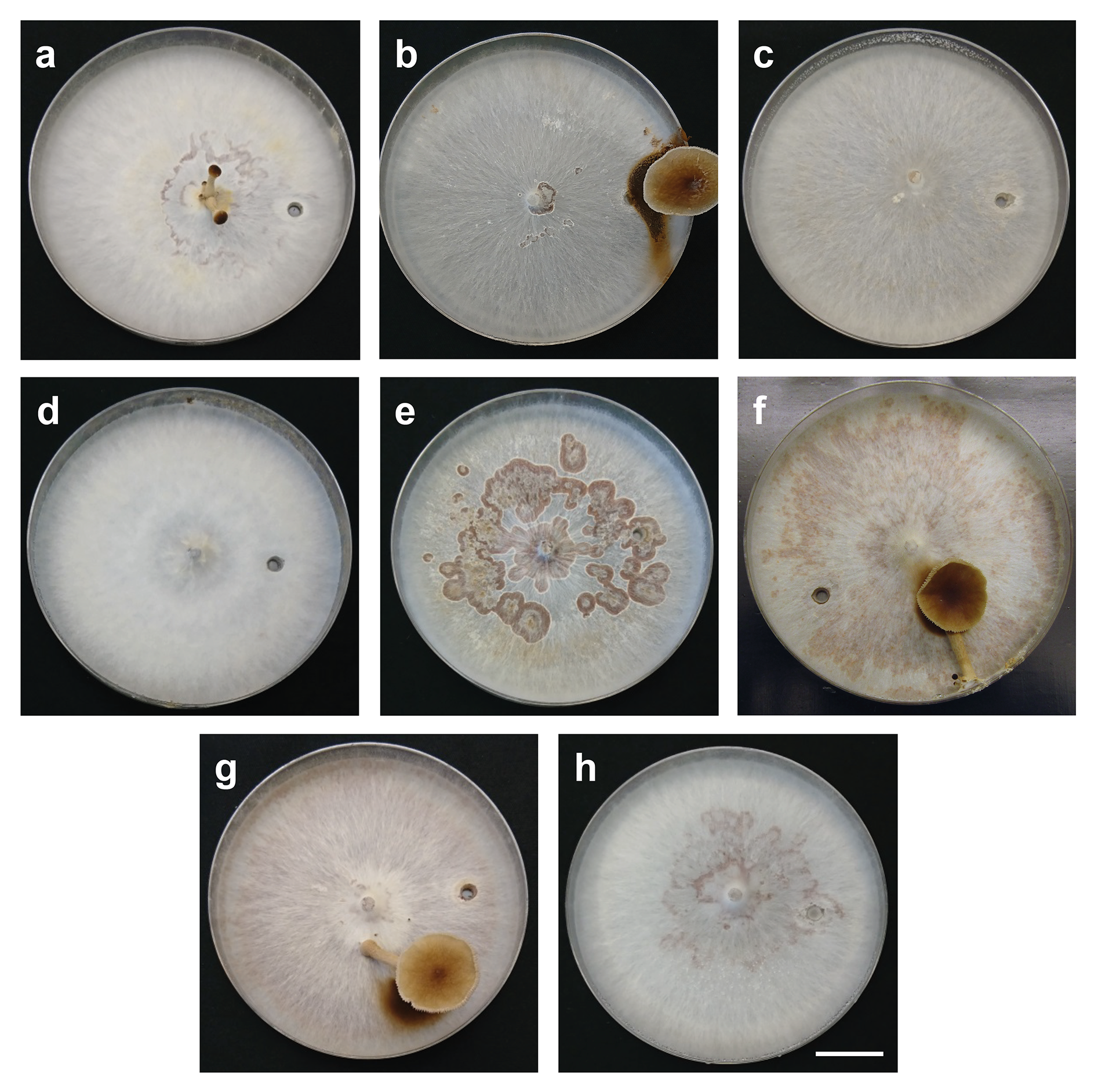

Supplement: Supplementary file 18 — High Resolution (TIF 3629 kb) [file 11557_2020_1599_MOESM9_ESM.tif]
